# Urbanization, air pollution, and water pollution: Identification of potential environmental risk factors associated with amyotrophic lateral sclerosis using systematic reviews

**DOI:** 10.3389/fneur.2023.1108383

**Published:** 2023-03-08

**Authors:** Daniel Saucier, Pierre Philippe Wilson Registe, Mathieu Bélanger, Colleen O'Connell

**Affiliations:** ^1^Faculty of Medicine and Health Sciences, Université de Sherbrooke, Sherbrooke, QC, Canada; ^2^Center de formation médicale du Nouveau-Brunswick, Moncton, NB, Canada; ^3^Stan Cassidy Center for Rehabilitation, Fredericton, NB, Canada; ^4^Department of Medicine, Dalhousie Medicine New Brunswick, Saint John, NB, Canada

**Keywords:** environmental risk factors, urbanization, air pollution, water pollution, amyotrophic lateral sclerosis, systematic review, diesel exhaust, heavy metals

## Abstract

**Introduction:**

Despite decades of research, causes of ALS remain unclear. To evaluate recent hypotheses of plausible environmental factors, the aim of this study was to synthesize and appraise literature on the potential associations between the surrounding environment, including urbanization, air pollution and water pollution, and ALS.

**Methods:**

We conducted a series (n = 3) of systematic reviews in PubMed and Scopus to identify epidemiological studies assessing relationships between urbanization, air pollution and water pollution with the development of ALS.

**Results:**

The combined search strategy led to the inclusion of 44 articles pertaining to at least one exposure of interest. Of the 25 included urbanization studies, four of nine studies on living in rural areas and three of seven studies on living in more highly urbanized/dense areas found positive associations to ALS. There were also three of five studies for exposure to electromagnetic fields and/or proximity to powerlines that found positive associations to ALS. Three case-control studies for each of diesel exhaust and nitrogen dioxide found positive associations with the development of ALS, with the latter showing a dose-response in one study. Three studies for each of high selenium content in drinking water and proximity to lakes prone to cyanobacterial blooms also found positive associations to ALS.

**Conclusion:**

Whereas markers of air and water pollution appear as potential risk factors for ALS, results are mixed for the role of urbanization.

## 1. Introduction

Amyotrophic lateral sclerosis (ALS) is a rare neurological disease characterized by selective loss of upper and lower motor neurons that ultimately results in the progressive loss of voluntary muscle function (1, 2). The diagnosis is based on clinical findings supported by electromyography and neuroimaging. People present with symptoms of muscle weakness, which can manifest as difficulty with mobility, and/or trouble with speech, swallowing, or breathing (3, 4). About one to two new cases per year are observed per 100,000 persons annually in most countries around the world with death usually resulting from respiratory failure within 3–5 years of symptom onset (5, 6). While specialized care can prolong survival and improve the quality of life of persons living with ALS (PALS) (7), no curative treatment exists and therapeutic options remain sparse as important gaps persist in the understanding of the etiology and heterogeneity of this motor neuron disease (MND) (8, 9). About 90% of cases are considered sporadic ALS (sALS) (10), meaning there is no known familial history and the cause is unknown. Furthermore, even when an ALS-related gene mutation is identified, this does not translate to certainty of developing ALS as an individual mutation can have multiple clinically distinct presentations among those affected (11). This led to a hypothesis that ALS is a complex genetic condition in which small contributions from various genetic and environmental factors interact to cause the disease (12). In fact, many genetic mutations associated with ALS have been identified in both the familial and sporadic form, suggesting these genetic variations might make people more susceptible to ALS with exogenous factors eventually triggering the neurodegeneration (13).

Because the ≥658 mutations in 126 genes identified to be linked to ALS only explain a small fraction of all ALS cases (14, 15), the role of genetic and environmental factors are generally considered to be of equal importance in the disease's etiology (8). However, while scientific advances in ALS genetics continue with the discovery of more genetic mutations associated with the disease and further understanding of their associated pathological mechanisms, the contribution of exogenous factors, including environmental factors, in the development of ALS remains largely unknown (15). In their review of factors potentially involved in the etiology of ALS, Oskarsson et al. (16) highlighted that risk factors beyond familial history likely do play a role in the development of ALS, but causation remains elusive with only male gender and smoking considered established risk factors. There was nevertheless an absence of studies regarding exposure to the surrounding environment of PALS, such as the local climate zone (17), air pollution, and waterways. However, biological plausibility exists for these potential environmental risk factors as uptake of heavy metals by motor neurons is common and higher risk of MND death have been observed in municipalities where industrial contaminants can be found in riverways (18, 19). As for air pollutants, fine particles from industrial activities and combustion can induce systematic inflammation when inhaled and lead to disruption of the blood-brain barrier's integrity (20).

Previous studies on the presence of environmental factors in proximity to one's residence also point to a possible relation to ALS. For example, a case-control study found that agricultural activity in the region, rather than rural residence itself, was significantly associated to ALS (21). Further research found that a greater number of PALS lived near an industrial factory as compared to age- and gender-matched controls (22). Regarding air pollution, associations between occupational diesel exhaust exposure and the development of ALS were observed in a large case-control study (23). As for water pollution, an ecological study found that proximity to water, especially lakes with toxic algae bloom indicators, appears to be associated with geographic areas with higher-than-expected ALS incidence (24). However, the growing literature on environmental factors in ALS, particularly exposures to the surrounding environment, such as urbanization and air and water pollution, has yet to be fully appraised and presented in a systematic review (15). An existing systematic review on exposure to rural environments only accounted for agricultural-related exposures and restricted inclusion of case-control and cohort studies only without providing an appraisal of risk of biases included in articles retained (25). Thus, the objective of this study is to identify, synthesize and appraise epidemiological studies relating to urbanization, air pollution and water pollution in relation to the development of ALS.

## 2. Methods

### 2.1. Literature search

A systematic review was conducted for each exposure of interest (*n* = 3). Indexed articles since database inception were searched in PubMed and Scopus using a combination of keywords/MeSH terminology and citation analysis (26), respectively. The last update of these searches was conducted on October 25, 2021. The complete search strategy for each exposure of interest can be found in the Supplementary material.

### 2.2. Selection process and eligibility criteria

Articles identified using the above search strategy were exported into Mendeley Desktop for further evaluation. Each article had their title and abstract screened for preliminary selection, followed by a full-text screening to confirm inclusion based on eligibility criteria prior to their detailed review and assessment. Manual screening of the reference lists of eligible studies was also conducted to identify additional studies that could have been missed by the initial search strategy. A study was eligible for inclusion in this review article if it satisfied all the following selection criteria:

- Peer-reviewed research article or research letter reporting original research.
- Non-experimental observational study (cohort studies, case control studies, population-based, ecological, administrative-based, or death registry).
- Cases of ALS are identified with ICD-8 code 348.0, ICD-9 code 335.20, or ICD-10 code G12.21 or through medical records, or are diagnosed by a physiatrist, neurologist, and/or specialist using electrodiagnostic or clinical diagnosis criteria [such as El Escorial (3)].
- Assesses an association between an environmental factor and ALS.

Experimental studies or studies pertaining only to genetic or prognostic factors were excluded. Case studies and conference abstracts were also not eligible. Studies with only mixed (e.g., ALS-FTD), restricted phenotype (ex. primary lateral sclerosis), familial or region-specific cases [e.g., Guam disease (27)] were also excluded.

### 2.3. Information extraction and assessment of included studies

Information on authors, year of publication, study region, study design, study participants, ALS diagnostic criteria, source of cases/exposed, source of controls/unexposed, environmental risk factors of interest, method of exposure ascertainment, and main findings pertaining to environmental risk factors of interest were extracted from the included articles by author DS. Extraction of information for articles excluded after full-text screening was limited to authors, year of publication, study region, title, and the main reason for exclusion. Included articles had their quality assessed by author DS using either the Newcastle-Ottawa Scale (separate assessment scales used for included cohort and case-control studies) (28) or a Modified Newcastle-Ottawa Scale as has been used in previous reviews (29, 30) for prevalence studies (used for included cross-sectional studies). For the Newcastle-Ottawa Scale, scores of 0–3, 4–6, and 7–9 were considered as low, moderate, and high quality, respectively, while cross-sectional studies judged with the modified version were considered of high risk of bias (<3 points) or of low risk of bias (≥3 points). Ecological studies did not have their quality assessed. The full Newcastle-Ottawa quality assessment scale guide for case control studies and for cohort studies, as well as the Modified Newcastle-Ottawa risk of bias scoring guide for cross-sectional studies, that were adapted for use in this study can be found in the Supplementary material. Author PPWR independently read, appraised, and extracted data from a subsample of over 10% of the included articles. Author agreement was over 95% with author MB resolving disagreements.

## 3. Results

### 3.1. Identification, screening, and summary characteristics of included studies

**Figure 1:**
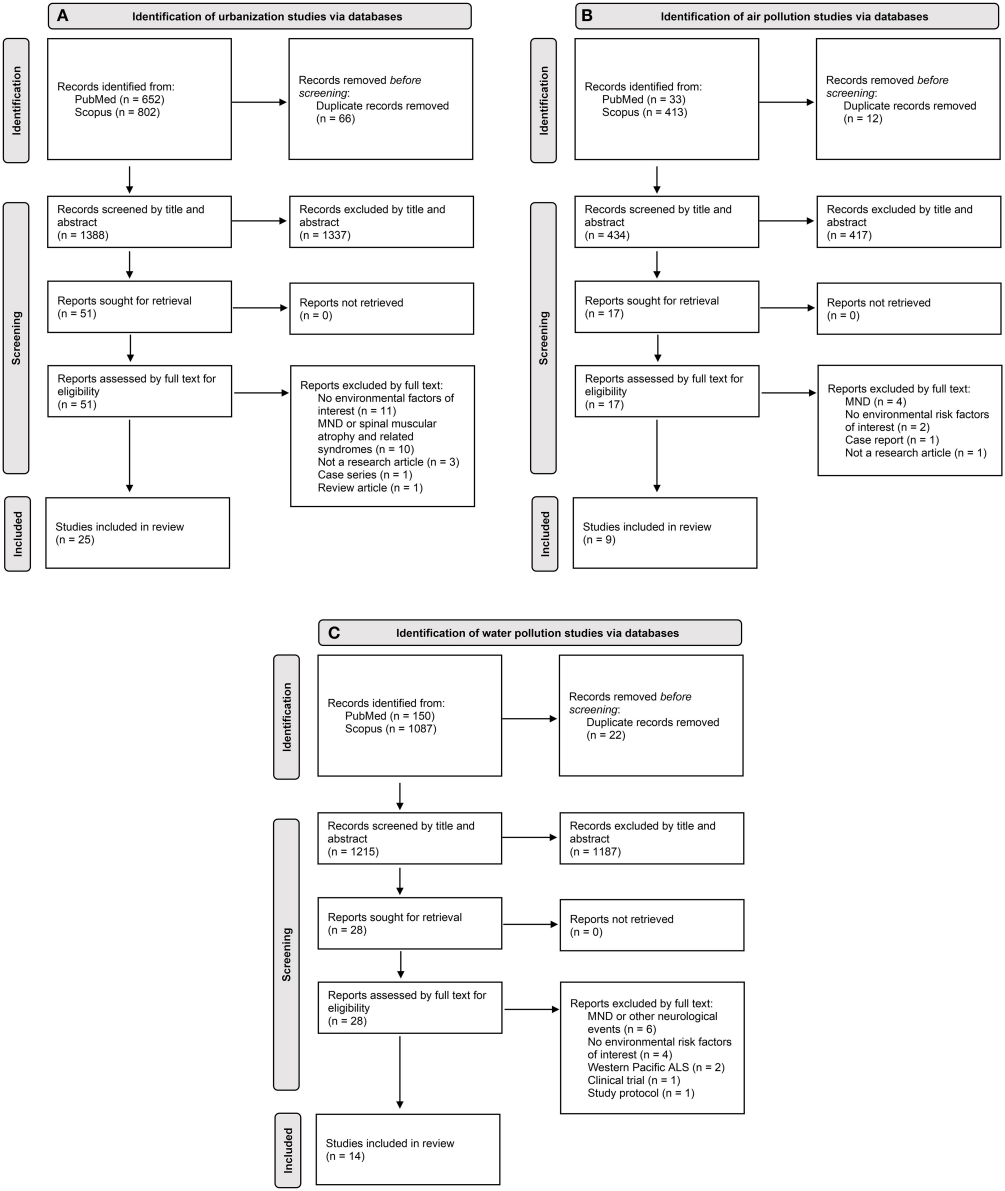
PRISMA (31) flowchart of study selection for each exposure of interest: **(A)** urbanization; **(B)** air pollution; **(C)** water pollution. Flow of information charts showing the number of unknown records identified through citation analysis on Scopus for each exposure of interest can be found in the Supplementary material.

The combined search strategy focused on identifying epidemiological studies that related to urbanization, air pollution and water pollution in the development of ALS and produced 1,388, 434 and 1,215 articles for each exposure of interest, respectively, after removal of duplicate citations (Figures 1A–C). These articles were screened based on their title and abstract. Studies pertaining to other diseases, experimental designs, genetic risk factors, environmental risk factors unrelated to those of interest and/or unrelated to disease etiology were deemed irrelevant to the goals of the review and thus excluded. The full papers of the remaining articles were read to further evaluate their eligibility and articles not fulfilling all selection criteria were excluded. No additional articles were included upon manual screening of the reference lists of eligible studies. Ultimately, the combined search strategy for urbanization, air pollution and water pollution in the development of ALS led to the inclusion of 25, 9, and 14 articles, respectively. Collectively, 44 of these articles were unique among the three systematic reviews.

Table 1 collectively summarizes the study characteristics of these 44 articles, while a further breakdown of these study characteristics, summarized by each of the three systematic reviews (urbanization, air pollution and water pollution) separately, is available in the Supplementary Table 1. Extracted information from the 25 included urbanization studies, nine included air pollution studies, and 14 included water pollution studies are presented in Tables 2–4, respectively. Further details on ALS diagnostic criteria, source of cases, source of controls and method of exposure ascertainment for each study is also respectively available in the Supplementary Tables 2–4. All studies were considered to be of either moderate or high quality or of low risk of bias. Specifically, none of the included case-control or cohort studies were assessed as being of low quality and neither of the two cross-sectional were assessed as having a high risk of bias. Individual scores by assessment item for the employed Newcastle-Ottawa scoring guides are also available for each study in the Supplementary Tables 5–7.

**Table 1:** Summary of study characteristics (region, method of exposure ascertainment, environmental factors of interest, study design, and Newcastle-Ottawa Scale quality assessment) for the 44 unique studies included from the combined search strategy.

**Region (*n* studies)**	**Method of exposure ascertainment (*n* studies)**	**Environmental factors of interest (*n* studies)**	**Study design (*n* studies)**	**NOS quality assessment^a^ (*n* studies)**
Italy (19^b^) USA (10) Ireland (4^b^) Netherlands (4^b^) France (3) Denmark (2) Argentina (1) Australia (1) China (1) Greece (1) Spain (1) UK (1)	Medical records (16) Questionnaire (16) Geospatial data (13) Census (11) Interview (9) Other (7) Water samples (5) Job-exposure matrix (4)	*Urbanization* Rural areas (9) Level of urbanization (7) Urban areas (7) Electromagnetic fields/powerlines (5) Agricultural land (4) Industrial activities (2) Waste disposal activities (2) Water/sewage treatment plants (2) Green areas (1) Petrol stations (1) Suburban areas (1) Traffic routes (1) *Air pollution* Diesel exhaust (4) Particulate matter (4) Hazardous air pollutants (3) Nitrogen oxides (3) Carbon monoxide (1) Hydrogen sulfide (1) Ozone (1) Particulates (1) Sulfur dioxide (1) Ultrafine particles (1) *Water pollution* Contaminated water (5) Lakes and cyanobacterial blooms (3) Proximity to water bodies (3) Source of drinking water (3) Water sports (1)	Case-control (24) Cohort (4) Cross-sectional (2) Ecological (14)	*NOS* High quality (17) Moderate quality (11) *Modified NOS* Low risk of bias (2) N/A (14)

NOS, Newcastle-Ottawa Scale; UK, United Kingdom; USA, United States of America.

a Score and classification of study's quality according to their assessment as described in Section 2.3.

b Includes two pooled European studies that included participants from Italy, Ireland, and the Netherlands.

**Table 2:** Description of 25 included urbanization studies.

**References**	**Region; Sample**	**Environmental factors of interest**	**Method of exposure ascertainment**	**Main findings pertaining to environmental risk factors of interest**	**NOS score (0–9)/modified NOS score (0–5)^a^**
**Case-control**					
Armon et al. (32)	USA 74 ALS cases 201 matched controls (age and sex)	Rural areas	Sequential questionnaire-interview	No significant difference for years lived in a rural community between ALS patients and controls (*p* = 0.621).	5 Moderate quality
Cruz et al. (33)	USA 174 ALS cases 348 matched controls (age, gender, and respondent type)	Rural areas	Structured in-person interview	There was no association between history of rural residence and ALS no matter the duration, accounting for matching on age, gender, and respondent type and adjusted for education, smoking, and residual confounding from age: Ever resided (OR = 0.8, 95% CI, 0.6–1.3); >0–5 years (OR = 1.0, 95% CI, 0.5–1.8); >5–20 years (OR = 0.8, 95% CI, 0.6–1.6); >20 years (OR = 0.7, 95% CI, 0.3–1.6).	7 High quality
Muddasir Qureshi et al. (34)	USA 95 ALS cases 106 matched controls (age and gender)	Rural areas, suburban areas, and urban areas	Comprehensive questionnaire	Geographical area was not associated with ALS (*p* = 0.81): Rural vs. urban (OR = 0.74, 95% CI, 0.28–1.93); Suburban vs. urban (OR = 0.81, 95% CI, 0.35–1.85).	6 Moderate quality
Furby et al. (21)	France 108 sALS cases 122 matched controls (age and sex)	Level of urbanization (inhabitants)	Structured questionnaire-interview	No significant difference was detected between patients and controls in the five identified levels of urbanization: under 1,000 inhabitants, between 1,000 and 5,000, between 5,000 and 8,000, between 8,000 and 100,000, and over 100,000 inhabitants (*p* = 0.707). Even when using a cut-off point of 8,000 inhabitants, there was no association between community size and ALS (OR = 1.350, 95% CI, 0.788–2.314). Consideration of community size in the first 20 years of life (OR = 1.338, 95% CI, 0.763–2.347) and 20 years before onset of disease or interview (OR = 1.349, 95% CI, 0.774–2.350) both also showed no association to ALS.	6 Moderate quality
Das et al. (35)	Italy 110 ALS cases 240 matched controls (age and sex)	Rural areas and urban areas	Open-ended structural interview	Rural dwellings were significantly associated with the development of ALS (OR = 1.99, 95% CI, 1.02–3.88).	6 Moderate quality
Yu et al. (22)	USA 66 ALS cases 66 matched controls (age and gender)	Agricultural land, industrial activities, and water/sewage treatment plants	Self-administered written questionnaire	No significant association between living near industry/sewage treatment plant/farm and ALS at four exposure windows: Exposure in the last 30 years (OR = 1.16, 95% CI, 0.41–3.30); Exposure in the last 10 years (OR = 1.15, 95% CI, 0.40–3.28); Exposure in the period from 30 years ago to 10 years ago (OR = 1.87, 95% CI, 0.69–5.11); Continuous exposure in the last 30 years (OR = 2.25, 95% CI, 0.69–7.27).	6 Moderate quality
Seelen et al. (36)	Netherlands 917 ALS cases 2,662 matched controls (age at symptom onset and gender)	Level of urbanization (addresses/km^2^)	Census (level of urbanization), medical records (ALS database for residential history)	Urbanization degree differed significantly between patients and controls (*p* < 0.001) with patients tending to live in more highly urbanized areas.	9 High quality
Vinceti et al. (37)	Italy 703 ALS cases 2,737 matched controls	Electromagnetic fields/powerlines	Geospatial data	Residence near high-voltage power lines yielding a magnetic field of ≥0.1 μT was not associated with an excess ALS risk using conditional logistic regression (OR = 0.65, 95% CI, 0.27–1.55) or unconditional logistic regression adjusted for age and sex (OR = 0.73, 95% CI, 0.16–3.31), nor was there a dose-response relationship.	8 High quality
Vinceti et al. (38)	Italy 703 ALS cases 2,737 matched controls (province of residence, sex, and year of birth)	Agricultural land	Geospatial data (land use), Revenue Agency (residential history)	Proximity to agricultural land (100 m radius) was not associated to odds of ALS (OR = 0.92, 95% CI, 0.78–1.09). Using long-term residence instead of current residence did not substantially change estimates.	8 High quality
Povedano et al. (39)	Spain Nested (cohort) 383 ALS cases 383 matched controls (age at diagnosis and sex)	Agricultural land, green areas, petrol stations, and traffic routes	Geospatial data	Statistically significant differences were found between the cases and the controls for distance to agricultural areas, residential streets, petrol stations and green areas. In multivariate models adjusted by sex, age, year of diagnosis, family history of disease, indicator of family in the database, and contextual deprivation index, <100 m distance to agricultural areas remained significant (Reference = >300 m, OR = 5.483, 95% CI, 1.279–25.23)	8 High quality
Peters et al. (40)	Europe (Ireland, Italy, and the Netherlands) 1,323 pooled ALS cases 2,704 pooled matched controls (age, geographic location, and sex)	Electromagnetic fields/powerlines	Job exposure matrix, structured questionnaire (occupational history)	Ever having had exposure to extremely low-frequency magnetic fields above background levels was significantly associated with ALS (OR = 1.16, 95% CI, 1.01–1.33) when unadjusted for other exposures, but not when adjusted for other exposures (OR = 1.10, 95% CI, 0.95–1.28). Both these models adjusted for sex, age, study center, education, smoking status, and alcohol drinking.	8 High quality
Filippini et al. (41)	Italy 95 ALS cases 135 matched controls (age, province of residence, and sex)	Agricultural land, electromagnetic fields/powerlines, and waste disposal activities	Self-administered questionnaire	Having lived near overhead powerlines (OR = 2.41, 95% CI, 1.13–5.12) was significantly associated with the odds of ALS. Ever lived in the countryside or had a farm (OR = 1.36, 95% CI, 0.78–2.37), ≥10 years lived in the countryside or had a farm (OR = 1.41, 95% CI, 0.79–2.51) and having lived near a waste incinerator (OR = 1.72, 95% CI, 0.50–5.87) or waste disposal site (OR = 0.57, 95% CI, 0.22–1.51) were all not associated to risk of ALS. All these models were adjusted by sex, age, and educational attainment.	6 Moderate quality
**Cohort**					
Luna et al. (42)^b^	France 312 ALS cases 714,012 (2000) to 738,766 (2012) persons	Electromagnetic fields/powerlines	Census, medical records (ALS register for residence), geospatial data	There was a significant progressive rise in the SIR of ALS with increases in the category of electromagnetic field exposure in both the non-cumulative (*p* = 0.0008) and cumulative (*p* = 0.0005) models. A relative risk of 1.55 (95% CI, 1.20–2.01) was estimated for every 1 V/m increase in exposure and a relative risk of 1.32 (95% CI, 1.13–1.52) was estimated for every 1 V/m/year increase in exposure.	8 High quality
**Cross-sectional**					
Bettini et al. (43)	Argentina 187 sALS cases	Rural areas and urban areas	Medical records	Eighty-four percent of the sALS patients came from urban areas.	3 Low risk of bias
Wei et al. (44)	China 1,131 ALS cases	Rural areas and urban areas	Face-to-face interviews	The percentage of patients from rural areas (64.6%) is higher than the estimated number of the rural population in the Sichuan province (55.1%, 2013 census).	4 Low risk of bias
**Ecological**					
Juvarra et al. (45)	Italy 78 ALS cases	Rural areas, urban areas	Medical records	The average annual incidence of ALS was 0.93 per 100,000 with no difference between rural and urban areas (0.91 vs. 0.97 per 100,000).	N/A
Kalfakis et al. (46)^c^	Greece 316 ALS cases	Rural areas	Census (percent rural), medical records (residence)	There was a significant positive correlation between the percentage of rural population and the number of ALS cases per prefecture as per Spearman's non-parametric method (rs = 0.396035, *p* < 0.05).	N/A
Bettoni et al. (47)	Italy 121 ALS cases	Rural areas, urban areas	Medical records	The average annual incidence of ALS was 0.98 per 100,000 with no difference between rural and urban areas (1.04 vs. 0.92 per 100,000).	N/A
Mandrioli et al. (48)	Italy 143 ALS cases	Flat areas, mountainous areas, and urban areas	Medical records	The mean incidence rate of ALS per 100,000 was higher in mountainous areas (3.67) with respect to urban (2.14) and flat ones (1.27).	N/A
Govoni et al. (49)	Italy 91 ALS cases	Level of urbanization (inhabitants)	Census (level of urbanization), medical records (residence)	There was no difference found between observed and expected cases of ALS in the four identified levels of urbanization (log-likelihood ratio test = 0.404, 3 degrees of freedom, 0.90 <*p* < 0.095). Incidence rate per 100,000: Villages <1,000 inhabitants and scattered houses (1.42); Small towns 1,000–5,000 inhabitants (1.77); Small towns 5,000–8,000 inhabitants (1.62); Towns> 100,000 inhabitants (1.67).	N/A
Scott et al. (50)	UK 368 ALS cases	Level of urbanization (population density)	Census (level of urbanization), medical records (ALS register for residence)	Age- and sex-adjusted relative risk of ALS in the south-east of England depended on population density (*p* = 0.01) with population density alone explaining 25% of the variance.	N/A
Boumédiène et al. (51)	France 177 ALS cases	Electromagnetic fields/powerlines, industrial activities (extractive, manufacturing, and storage of pollutants industries), waste disposal activities, and water/sewage treatment plants	Census (communes), geospatial data	The SIR of ALS in communes with a paper and cardboard industry was 1.28 (95% CI, 1.01–1.60). Communes with disposal activities and companies involved in cleaning and/or discharge also had meaningful results with an SIR of 1.39 (95% CI, 1.00–1.87). Spatial analysis by distance to high-voltage facilities in 100 m buffer intervals up to 1 km revealed an SIR of 1.54 (1.08–2.10) for <400 m.	N/A
Mandrioli et al. (52)	Italy 347 ALS cases	Level of urbanization (population density)	Census (level of urbanization), medical records (address)	The mean incidence rate of ALS in municipalities with a population density of <50 inhabitants/km^2^ was 3.27 per 100,000, whereas in municipalities with a population density of ≥ 50 inhabitants/km^2^ mean incidence rate was 2.55 per 100,000 with an increasing gradient from areas of low density to areas of higher density.	N/A
Rooney et al. (53)	Ireland 1,638 ALS cases	Level of urbanization (population density)	Census, medical records (ALS register for residence)	Results from Bayesian spatial auto-regression were conflicting, while a weighted linear regression approach of post Bayesian smoothing indicated a weak, albeit statistically significant, relationship between ALS and population density (Regression coefficient = 0.22, 95% CI, 0.211–0.234).	N/A
Rooney et al. (54)	Ireland 1,701 ALS cases	Level of urbanization (population density)	Census (level of urbanization), medical records (ALS register for residence)	Population density did not explain the spatial variation of ALS risk (Regression coefficient = −3.1, 95% CI, −18.8–11.6).	N/A

ALS, amyotrophic lateral sclerosis; CI, Confidence Interval; N/A, not applicable; NOS, Newcastle-Ottawa Scale; OR, odds ratio; sALS, sporadic amyotrophic lateral sclerosis; SIR, standardized incidence ratio; UK, United Kingdom; USA, United States of America.

a Score and classification of study's quality according to their assessment as described in Section 2.3.

b The geo-epidemiological population study had a non-classical cohort design, similar to a developmental research or natural history study.

c Although the study had a case-control design, the geographical analysis was ecological in nature and did not utilize the controls as a comparison group for the urbanization risk factor of interest.

**Table 3:** Description of nine included air pollution studies.

**References**	**Region; Sample**	**Environmental factors of interest**	**Method of exposure ascertainment**	**Main findings**	**NOS score (0–9)/modified NOS score (0–5)^a^**
**Case-control**					
Pamphlett and Rikard-Bell (55)	Australia 611 sALS cases 755 controls	Occupation exposure to diesel exhaust	Self-reported questionnaires	Only truck driving in males was statistically significant as an individual occupational title with increased odds of sALS (OR = 2.36; 95% CI, 1.21–4.59), while 10 out of the 11 occupational titles in the study with potential exposure to diesel exhaust were associated with an increased odds of sALS according to previous epidemiological studies.	6 Moderate quality
Malek et al. (56)	USA 66 sALS cases 66 matched controls (age, race, and sex) (*n* = 47 pairs in 1999) (*n* = 51 pairs in 2002) (*n* = 50 pairs in 2005)	HAPs (aromatic solvents, metals, organic/chlorinated solvents, other HAPs, and pesticides)	Census (linking), detailed questionnaire (residential history), geospatial data (HAPs)	The odds of ALS was significantly decreased in 2005 (OR = 0.29; 95% CI, 0.10–0.90) for those exposed to upper quartiles of organic/chlorinated solvents after adjustment for education and smoking. Only those exposed to upper quartiles of aromatic solvents had a significantly increased odds of ALS in 1999 (OR = 4.27, 95% CI, 1.09–16.79) and in 2002 (OR = 5.03, 95% CI, 1.29–19.53) in the final multivariate models adjusted for education, smoking and other exposure groups. Metals, pesticides, and other HAPs all had no significant association to the risk of ALS for the years 1999, 2002, and 2005 in all models.	7 High quality
Seelen et al. (36)	Netherlands 917 ALS cases 2,662 matched controls (age at symptom onset and gender)	Nitrogen oxides (NO_2_ and NO_X_) and particulate matter (PM_10_, PM_coarse_, and PM_2.5_)	Geospatial data (LUR models), medical records (ALS database for residential history)	The odds of ALS was significantly increased for individuals in the upper exposure quartile (Q4) of PM_2.5_ absorbance (OR = 1.67, 95% CI, 1.27–2.18), NO_2_ (OR = 1.74, 95% CI, 1.32–2.30), and NO_X_ concentrations (OR = 1.38, 95% CI, 1.07–1.77) in conditional logistic regression models adjusted for gender, age, educational level, current smoking status, current alcohol consumption, BMI, and area-level SES. These associations remained significant in two-pollutant models adjusted for PM_2.5_ or NO_2_. These results, except for NO_X_, remained significant in single-pollutant models after adjusting additionally for urbanization degree, with PM_2.5_ absorbance and NO_2_ showing linear trends (*p* = 0.002 and 0.03, respectively).	9 High quality
Dickerson et al. (23)	Denmark 1,639 ALS cases 151,975 matched controls (birth-year and sex)	Occupation exposure to diesel exhaust	Danish Pension Fund (occupational history), job exposure matrix	Using a 10-year lag period, diesel exhaust exposure was positively associated with ALS among men who had ever been exposed (aOR = 1.20, 95% CI, 1.05–1.38) in models adjusted for socioeconomic status and residential location. For men with >50% probability of diesel exposure, there was a significant positive association between ALS and the highest-quartile of exposure during the 5-year (aOR = 1.35, 95% CI, 1.07–1.70) and 10-year (aOR = 1.41, 95% CI, 1.11–1.79) lag periods, along with showing linear trends, in models adjusted for socioeconomic status and residential location. These associations were not seen in women.	7 High quality
Povedano et al. (39)	Spain Nested (cohort) 383 ALS cases 383 matched controls (age at diagnosis and sex)	CO, HAPs (benzene, benzopyrene, metals), H_2_S, nitrogen oxides (NO_2_ and NO), O_3_, particulate matter (PM_10_ and PM_2.5_), and SO_2_	Geospatial data, medical records (ALS database for residential history)	Statistically significant differences were found between the cases and the controls for exposure levels of some atmospheric contaminants (SO_2_, O_3_, and benzene) and metals (Pb, Ni, and Cd); and in the levels of arsenic. In multivariate models adjusted by sex, age, year of diagnosis, family history of disease, indicator of family in the database and contextual deprivation index, only NO_2_ was significantly associated with the occurrence of ALS. NO_2_ presented an increasing gradient for the OR: Quartile 1 (reference); Quartile 2 (OR = 1.872, 95% CI, 1.487–2.023), Quartile 3 (OR = 2.047, 95% CI, 1.698–2.898), and Quartile 4 (OR = 2.703, 95% CI, 1.265–3.255).	8 High quality
Visser et al. (57)	Europe (Ireland, Italy, and the Netherlands) 1,252 pooled ALS cases 2,590 pooled matched controls (age, gender, and residency)	Occupation exposure to diesel exhaust, HAPs (asbestos and polycyclic aromatic hydrocarbons) and particulates (animal contact, endotoxin, organic dust, and silica)	Face-to-face interviews (Ireland and Italy), structured questionnaire (self-report in Netherlands), job exposure matrix	There was a significant positive linear association between occupational diesel motor exhaust and ALS in a logistic regression model adjusted for age, education, smoking, alcohol, and gender (*p* = 0.03 for trend on a continuous scale). Occupational exposure to organic dust (*p* = 0.03) and silica (*p* = 0.01) were also significantly associated to ALS on the continuous scale, while only silica had a significant OR for both low (OR = 2.24; 95% CI, 1.50–3.35) and high (OR = 2.13; 95% CI, 1.42–3.20) exposures relative to those never exposed. Exposure to other particulates (animal contact, asbestos, and endotoxin) and polycyclic aromatic hydrocarbons were not significantly associated with ALS on a continuous scale.	8 High quality
Bellavia et al. (58)	Denmark Nested (cohort) 1,086 ALS cases 111,507 matched controls (birth year and sex)	Occupation exposure to diesel exhaust	Danish Pension Fund (occupational history), job exposure matrix	No significant association between diesel exhaust and ALS (OR = 0.79, 95% CI, 0.79–1.28).	7 High quality
Filippini et al. (59)	Italy 52 ALS cases 80 controls	Particulate matter (PM_10_)	Geospatial data, medical records (address)	Using fixed cutpoints at 5, 10, and 20 of the annual median PM_10_ levels, and compared with exposure <5 μg/m^3^, there was no excess ALS risk at 5–10 μg/m^3^ (OR = 0.87, 95% CI, 0.39–1.96), 10–20 μg/m^3^ (OR = 0.94, 95% CI, 0.24–3.70), and ≥20 μg/m^3^ (OR = 0.87, 95% CI, 0.05–15.01). Using maximum PM_10_ concentrations, excess ALS risk for subjects exposed at 10–20 μg/m^3^ compared with those exposed <10 μg/m^3^ was statistically unstable (OR = 4.27, 95% CI, 0.69–26.51).	8 High quality
Yu et al. (60)	Netherlands 1,636 ALS cases 4,024 matched controls (age and sex)	Nitrogen oxides (NO_2_ and NO_X_), particulate matter (PM_10_, PM_coarse_, PM_2.5_, and PM elemental constituents) and ultrafine particles	Geospatial data, medical records (ALS database for residential history)	Most air pollutants had significant associations with the odds of ALS with the strongest associations being for NO_2_ (OR = 1.25, 95% CI, 1.15–1.34), PM2.5 absorbance (OR = 1.19, 95% CI, 1.10–1.28), and NO_X_ (OR = 1.14, 95% CI, 1.07–1.22) in single-pollutant models adjusted for sex, age, education level, BMI, smoking status, alcohol consumption, and area SES. Ultrafine particles were also significantly associated with the odds of ALS (OR = 1.11, 95% CI, 1.05–1.16). For particle elements, road traffic non-tail pipe emissions of Cu, Fe, Ni, S, Si, and V were associated with significantly higher ORs for ALS in both PM_2.5_ and PM_10_ fractions. Independent associations between NO_2_, PM_10_Si, and the odds of ALS were found with most air pollutants reduced toward the null after correction for NO_2_ in two-pollutant models.	8 High quality

ALS, amyotrophic lateral sclerosis; aOR, adjusted odds ratio; BMI, body mass index; Cd, cadmium; CI, Confidence Interval; CO, carbon monoxide; Cu, copper; Fe, iron; H_2_S, hydrogen sulfide; HAPs, hazardous air pollutants; Ni, nickel; NO, nitrogen oxide; NO_2_, nitrogen dioxide; NOS, Newcastle-Ottawa Scale; NO_X_, nitrogen oxides; OR, odds ratio; Pb, lead; PM, particulate matter; PM_2.5_, particulate matter (fine); PM_10_, particulate matter; S, sulfur; sALS, sporadic amyotrophic lateral sclerosis; SES, socioeconomic status; Si, silicon; SO_2_, sulfur dioxide; USA, United States of America; V, vanadium.

a Score and classification of study's quality according to their assessment as described in Section 2.3.

**Table 4:** Description of 14 included water pollution studies.

**References**	**Region; Sample**	**Environmental factors of interest**	**Method of exposure ascertainment**	**Main findings**	**NOS score (0–9)/modified NOS score (0–5)^a^**
**Case-control**					
Vinceti et al. (61)	Italy 41 ALS cases 82 matched controls (sex and year of birth)	Contaminated water (high Se content)	Municipal Water Supply Agency, questionnaire-interview, water samples	Consumption of drinking water containing ≥1 μg/l of inorganic Se was significantly associated with ALS (RR = 5.4, 95% CI = 1.1–26) even after adjustment for all potential confounders (educational attainment level and exposures to pesticides, industrial chemicals, and electromagnetic fields). Greater amounts of cumulative inorganic Se intake were also shown to progressively increasing the effect on relative risk for ALS.	7 High quality
Das et al. (35)	Italy 110 ALS cases 240 matched controls (age and sex)	Source of drinking water	Open-ended structural interview	Source of drinking was not associated with ALS: Tube well (OR = 1.41, 95% CI, 0.79–2.54), well water (OR = 0.99, 95% CI, 0.46–2.14), ponds (OR = 0.46, 95% CI, 0.13–1.65), and corporation supplied water (OR = 1.26, 95% CI, 0.80–2.67).	6 Moderate quality
Andrew et al. (62)	USA 295 ALS cases 224 controls	Proximity to water bodies, water sports	Questionnaire	Increased odds of ALS associated with ever full-time within 2 miles of a waterbody (OR = 1.59, 95% CI, 1.05–2.42), and frequent participation in water sports, specifically water-skiing (OR = 3.89, 95% CI, 1.97–8.44) and boating, sailing, or kayaking (OR = 1.51, 95% CI, 1.01–2.28). Water-skiing retained independent statistical significance in a composite model containing age, gender, and smoking status (adjusted OR = 3.71, 95% CI, 1.80–8.47).	6 Moderate quality
Filippini et al. (41)	Italy 95 ALS cases 135 matched controls (age, province of residence, and sex)	Proximity to water bodies	Self-administered questionnaire	Having ever lived <3 km from water bodies, adjusted by sex, age, and educational attainment, was significantly associated with the odds of ALS (OR = 1.83, 95% CI, 1.04–3.21).	6 Moderate quality
Filippini et al. (63)	Italy 95 ALS cases 135 matched controls (age, province of residence, and sex)	Source of drinking water	Self-administered questionnaire	Both current use of any private well water (OR = 1.91, 95% CI, 0.76–4.79) or ever use a private well/fountain for drinking water (OR = 1.38, 95% CI, 0.73–2.27) were not significantly associated with the odds of ALS in models adjusted by sex, age, and educational attainment. Main source of drinking water was also not associated with the odds of ALS in models adjusted by sex, age, and educational attainment: No preference (reference); municipal water (OR = 0.55, 95% CI, 0.28–1.07); private wells (OR = 1.44, 95% CI, 0.29–7.03); bottled water (OR = 1.24, 95%CI, 0.62–2.44).	6 Moderate quality
Fiore et al. (64)	Italy 703 ALS cases 2,737 matched controls (province of residence, sex, and year of birth)	Proximity to water bodies	Geospatial data (water bodies), governmental data (addresses)	There was no significant association between odds of ALS related to water bodies within 100 m from subject's residence at the time of diagnosis (OR = 1.41, 95% CI, 0.72–2.74) or from the subject's residence in the former 30 years (OR = 1.31, 95% CI, 0.57–2.99).	9 High quality
Stipa et al. (65)	Italy 33 ALS cases 35 matched controls (category of municipality residence)	Source of drinking water	Questionnaire, face-to-face interview	Those that were chronically exposed to raw water (untreated) had a significantly increased odds of ALS (OR = 6.55, 95% CI, 2.24–19.12), whereas those that used a private well were not (OR = 2.64, 95% CI, 0.59–11.83). This association remained significant even after adjustment for age, sex, and heavy physical activities (OR = 4.74, 95% CI, 1.33–16.85).	5 Moderate quality
**Cohort**					
Vinceti et al. (66)	Italy Prospective 5,182 residents of Reggio Emilia municipality exposed to drinking water with high selenium content Remaining municipal population (unexposed)	Contaminated water (high Se content)	Interviews with next of kin, list of the 1980 AGAC (municipal water agency) subscribers	During the follow-up, four cases of sALS were diagnosed with all of them occurring in cohort members with the longest ascertainable period of exposure. The SIR was 4.22 (95% CI, 1.15–10.80) in comparison to the reference group with a higher SIR upon limiting the analysis to a sub-cohort of those with the longest ascertainable exposure period (SIR = 8.90, 95% CI = 2.43–22.79). In both analyses, females seemed to have a stronger effect with a SIR of 9.77 (95% CI, 2.02–28.56) for the total cohort and of 21.36 (95% CI, 4.41–62.44) for the sub-cohort as opposed to a SIR of 1.56 (95% CI = 0.04–8.70) for the total cohort and of 3.24 (95% CI, 0.08–18.03) for the sub-cohort in males.	7 High quality
Bove et al. (67)	USA Retrospective 154,932 marine and naval personnel exposed to contaminated drinking water (Camp Lejeune) 154,969 marine and naval personnel unexposed to contaminated drinking water (Camp Pendleton)	Contaminated water (solvents)	Water samples	Comparisons of ALS mortality adjusted by sex, race, rank, and education, 10-year lag, between the two cohorts did not reveal an elevated HR for ALS (0.83, 95% CI, 0.47–1.48).	5 Moderate quality
Vinceti et al. (68)	Italy Prospective 2,065 Rivalta residents with the highest and longest exposure to selenium from 1974 to 1985 Remaining 95,715 people who resided in the Reggio Emilia municipality from 1974 to 1985 (unexposed)	Contaminated water (high Se content)	Door-to-door and postal surveys (source of water), health unit/registry office (residential history), subscribers to the 1974 Municipal Water Supply Agency (for exposed)	From 1986 to 2015, a crude incidence rate of 14 cases per 100,000 person-years was identified in the exposed cohort as opposed to a crude incidence rate of five cases per 100,000 person-years in the unexposed cohort. Poisson regression analysis, adjusting for age, sex, and calendar year, revealed an overall IRR for ALS of 2.8 (95% CI, 1.3–6.0) with a substantially stronger IRR of 8.2 (95% CI, 1.8–14.3) in 1986–1994 than in 1995–2015 (IRR = 1.5, 95% CI, 0.5–4.7). Stronger effects among women were noted with an IRR of 5.1 (95% CI, 1.8–14.3) as opposed to an IRR of 1.7 (95% CI, 0.5–5.4) in men.	8 High quality
**Ecological**					
Caller et al. (69)^b^	USA 278 sALS cases	Lakes and cyanobacterial blooms	Census (boundaries), geospatial data, medical records (address), water samples	The odds ratio of developing ALS for persons living within 0.5 miles of a lake with current or past cyanobacterial blooms, compared to the odds for persons living further away from cyanobacterial blooms, was 2.32 (95% CI, 1.42–3.80).	N/A
Torbick et al. (24)^b^	USA 754 ALS cases	Lakes and cyanobacterial blooms	Questionnaire (residence), geospatial data (remote sensing), water samples	All 754 ALS cases had at least 1 lake within 30 km, while 709 ALS cases had at least one lake within 10 km. The final selected model of lake attributes indicated Secchi depth (30 km; OR = 0.40, 95% CI, 0.249–0.614) and total nitrogen (30 km; OR = 2.42, 95% CI, 1.460–4.124) are significantly associated with higher odds of belonging to a census tract identified as an ALS hot spot.	N/A
Torbick et al. (70)	USA 347 ALS cases	Lakes and cyanobacterial blooms	Questionnaire (residence), geospatial data (remote sensing), water samples	There was a 48% increase in ALS risk noted when average phycocyanin concentration (proxy measure for reoccurring seasonal cyanobacterial harmful algal blooms) is 100 μg/L.	N/A
Tesauro et al. (71)	Italy 106 ALS cases identified in a previous study (72)	Contaminated water (metals)	Water samples	The study was prompted by a higher incidence of sALS in the Briga area between 2002 and 2012 in comparison with the entire province, as well as environmental contamination in the area (72). Results of the monitoring campaign carried out in the period 1989–1995 showed high metal contamination (Cr[III], Cr[IV], Cu, Ni, and Zn) in surface waters with severe metal contamination (Cr[III], Cu, Ni, and Zn) also found at points sources and discharging in the fresh surface waters of the Briga area. Site-specific groundwater metal contamination (Al, Cu, Mn, and Ni) was found even if diffuse contamination of groundwater in the Briga area was not demonstrated.	N/A

Al, aluminum; ALS, amyotrophic lateral sclerosis; CI, Confidence Interval; Cr, chromium; Cu, copper; HR, hazard ratio; IRR, incidence rate ratio; Mn, manganese; N/A, not applicable; Ni, nickel; NOS, Newcastle-Ottawa Scale; OR, odds ratio; RR, relative risk; sALS, sporadic amyotrophic lateral sclerosis; Se, selenium; SIR, standardized incidence ratio; USA, United States of America; Zn, zinc.

a Score and classification of study's quality according to their assessment as described in Section 2.3.

b Although the studies had calculation of odds ratios through separation of cases into two groups, such as according to ALS hotspot membership, they were classified as ecological in nature given their eco-epidemiological modeling approach.

### 3.2. Qualitative synthesis of urbanization factors associated with the development of ALS

#### 3.2.1. Rural areas, agriculture land, urban areas, and level of urbanization

A total of nine papers studied rural areas as a risk factor for ALS. Of those, two found a significant positive association or correlation between ALS and rural areas (35, 46) and two noted a higher percentage of patients from rural areas than the estimated number of the rural population (43, 44). The other five studies found no association (32–34, 45, 47), including one case-control study that considered previous history of rural residence for various durations *via* structured interviews (33). Among the four studies noting a positive relation to ALS, over-representation of elementary occupations, such as laborers in agriculture, mining, manufacturing and transport (43), or farmers (46) were noted. Previous exposure to pesticides was also noted to be either higher among ALS cases in comparison to controls (35) or those PALS with a history of exposure mainly came from rural areas (44). While one study noted a higher rate of ALS (3.67 per 100,000) for those residing in mountainous areas, which according to the authors may be explained by the high prevalence of agricultural work in those areas in comparison to urban or flat areas (48), only one case-control study found a significant positive association between proximity to agricultural areas and the odds of ALS (OR = 5.483, 95% CI, 1.279–25.23) (39). No association between agricultural land and ALS was found in the three other case-controls studies evaluating this exposure of interest (22, 38, 41).

While the above studies provide some evidence to suggest rural area involvement in ALS, whether directly or indirectly as a factor, there is also evidence to suggest the contrary. Although living in suburban (34) or urban areas (34, 35, 43–45, 47, 48) were not associated with the risk of ALS in any of the included studies, three out of the seven studies categorizing place of residence into various levels of urbanization found a significant positive association between those living in more highly urbanized/dense areas and the risk of ALS (36, 50, 53). As opposed to the often poorly defined or absent definitions of rural vs. urban areas in the other studies, level of urbanization in these studies were determined by set cut-offs using number of inhabitants, population density or addresses/km^2^. However, an inverse association (52) or no association at all (21, 49, 54) between level of urbanization and ALS were found in the other four studies. Additionally, of the three studies identifying a positive association between ALS and highly urbanized areas, two were ecological (50, 53) and the case-control study was primarily focused on air pollution (36). Degree of urbanization was mainly included in the study as a control for urban, peri-urban, and rural differences in lifestyle and other environmental factors. Overall, evidence for the above urbanization factors is mixed at best among the included studies.

#### 3.2.2. Electromagnetic fields and high-voltage infrastructure in the surrounding area

After rural areas, urbans areas, and level of urbanization, electromagnetic fields and/or proximity to powerlines was the next most studied urbanization risk factor among included studies (37, 40–42, 51). Once adjusted for confounders, only three out of five of these studies found a significant positive association between ALS and electromagnetic fields and/or high-voltage infrastructure such as powerlines (41, 42, 51). However, one of these three positive studies used an ecological approach (51), while the positive case-control study had under 100 ALS cases and was subject to recall bias as it was the only study of five to ascertain the exposure with a self-administered questionnaire (41). The study finding no association at all between ALS and electromagnetic fields/powerlines had a more substantial number of cases (*n* = 703) and controls (*n* = 2,737) and utilized geospatial data (37).

#### 3.2.3. Industrial-related activities in the surrounding area

Various industrial related activities were also studied in the included urbanization papers, albeit with mixed results. An ecological study noted that communes with a paper or cardboard industry (manufacturing industries) had an elevated standardized incidence ratio (SIR) of ALS of 1.28 (95% CI, 1.01–1.60), as well as communes with waste disposal activities and companies involved in cleaning and/or discharge (SIR = 1.39, 95% CI, 1.00–1.87), while those communes with extractive industries, storage of pollutants industries or water treatment plants did not have an elevated SIR (51). In contrast, a case-control study using a self-administered questionnaire reported that having lived near a waste incinerator (OR = 1.72, 95% CI, 0.50–5.87) or waste disposal site (OR = 0.57, 95% CI, 0.22–1.51) were not associated to ALS (41). Finally, a case-control study of 66 matched pairs found no significant association between living near industry/sewage treatment plant/farm and ALS at four exposure windows: exposure in the last 30 years (OR = 1.16, 95% CI, 0.41–3.30); exposure in the last 10 years (OR = 1.15, 95% CI, 0.40–3.28); exposure in the period from 30 years ago to 10 years ago (OR = 1.87, 95% CI, 0.69–5.11); continuous exposure in the last 30 years (OR = 2.25, 95% CI, 0.69–7.27) (22). However, this may be a statistical power issue given the rather small sample size.

#### 3.2.4. Petrol stations, traffic routes, and green areas

Once adjusted for various potential confounders, there were no significant difference between the individuals with ALS and controls for distance to the nearest petrol stations, residential streets, and green areas in a nested case-control study by Povedano et al. in Spain (39).

### 3.3. Qualitative synthesis of air pollution factors associated with the development of ALS

#### 3.3.1. Residential exposure to atmospheric pollutants

Malek et al. (56) were the first to investigate residential exposure to ambient air pollution in relation to ALS. The matched (age, sex, and race) case-control study looked at U.S. Environmental Protection Agency National-Scale Air Toxics Assessment data for the years 1999, 2002, and 2005 to estimate odds ratio of developing ALS among persons living in census tracts with greater exposure to air pollutants (3rd and 4th quartiles) relative to those living in a census tract with lesser exposure (1st and 2nd quartiles). In the final adjusted multivariate models, only those exposed to upper quartiles of aromatic solvents had a significantly increased odds of ALS in 1999 (47 pairs, OR = 4.27, 95% CI, 1.09–16.79) and in 2002 (51 pairs, OR = 5.03, 95% CI, 1.29–19.53). Metals, pesticides, and other hazardous air pollutants all had no significant association to the odds of ALS for the years 1999, 2002, and 2005 in all models.

Much larger case-control studies have since been conducted to investigate the effect of long-term residential exposure to air pollution in relation to ALS (36, 39). In the Netherlands (36), the odds of ALS was significantly increased for individuals in the upper exposure quartile of fine particulate matter (PM_2.5_) absorbance (OR = 1.67, 95% CI, 1.27–2.18), nitrogen dioxide (NO_2_; OR = 1.74, 95% CI, 1.32–2.30), and nitrogen oxides (NO_X_) concentrations (OR = 1.38, 95% CI, 1.07–1.77) in the fully adjusted models. PM_2.5_ absorbance and NO_2_ remained significant after adjusting additionally for urbanization degree and showed linear trends (*p* = 0.002 and 0.03, respectively). Such findings were also observed in a follow-up research letter to this Dutch study (60). Additional analyses included further analysis of PM_2.5_ and particulate matter (PM_10_) elemental fractions of road traffic non-tail pipe emissions, which were associated with significantly higher ORs for ALS in copper, iron, nickel, sulfur, silicon (Si) and vanadium particulate and fine particulate elemental constituents. However, only the independent associations between NO_2_, PM_10_Si, and the odds of ALS remained after correction for NO_2_ in two-pollutant models. Ultrafine particles were not associated with ALS. In both the original study and the follow-up research letter, PM_10_ itself was not associated with the odds of ALS (36, 60); a finding which was also reported in a small case-control study in Italy (59).

A case-control study in Spain also reported no association between PM_10_ and ALS, in addition to PM_2.5_. Benzopyrene, a hazardous air pollutant, as well as carbon monoxide (CO), hydrogen sulfide and nitrogen oxide (NO) were also not associated with ALS. Statistically significant differences between the cases and the controls were seen, however, for exposure levels of some atmospheric contaminants [sulfur dioxide [SO_2_], ozone [O_3_] and benzene], metals (Pb, Ni, and Cd) and in the levels of arsenic (39). However, only NO_2_ was significantly associated with the occurrence of ALS in fully adjusted multivariate models. NO_2_ also presented an increasing gradient for the OR (Reference = 1st quartile) of ALS: Quartile 2 (OR = 1.872, 95% CI, 1.487–2.023), quartile 3 (OR = 2.047, 95% CI, 1.698–2.898), and quartile 4 (OR = 2.703, 95% CI, 1.265–3.255).

Overall, many high-quality studies, all using a case-control design and accounting for various factors, identified a positive association between residential exposure to atmospheric pollutants and the odds of ALS. NO_2_ was not only significantly associated with increased odds of ALS in all three papers that studied this pollutant (36, 39, 60), but also showed a dose-response with an increasing gradient for the OR of ALS as the quartile of exposure increased (39). There is strong evidence for NO_2_ as a potential risk factor for ALS. As for particulate matter, PM_10_ itself was not associated with ALS in all four papers studying it (36, 39, 59, 60), while the positive association between PM_2.5_ and ALS seen in the two Dutch studies (36, 60) was not reported in a separate population from Italy (39). Finally, emissions with metals in their composition, particularly nickel, were shown to have an association with the development of ALS in two studies based on distinct populations (39, 60).

#### 3.3.2. Occupational exposure to diesel exhaust and other work-related particulates

Of the 4 studies that assessed occupation exposure to diesel exhaust as a risk factor, 3 of them found it to be positively associated with ALS. Pamphlett and Rikard-Bell (55) reported that, in comparison to control individuals, sALS males were more likely to be in major occupation groups requiring manual labor and tasks, such as technicians and trade workers, machine operators and drivers and laborers (55). Among these manual labor and task occupations, truck driving was the only individual occupation (no major grouping of occupations) in males that was statistically significant as an occupational title with increased odds of sALS (OR = 2.36; 95% CI, 1.21–4.59). The authors considered diesel exhaust exposure to be the most likely environmental factor in truck drivers responsible for this association as 91% (10 out of 11) of the occupational titles in this study with potential exposure to diesel exhaust have been associated with an increased odds of sALS according to previous epidemiological studies. This finding is further supported by two case-control studies that used a job exposure matrix to investigate the relationship between occupational diesel exhaust exposure and ALS (23, 57). As reported by Dickerson et al. (23), Danish men in the highest quartile of cumulative diesel exhaust exposure intensity in 10-year-lag analyses had a 41% increased odds of ALS in adjusted analysis with overall trends for 5- and 10-year lags also being significant. While the same was not seen in women, Visser et al. (57) in a pooled European study reported a significant positive association between occupational diesel motor exhaust and ALS (*p*-value of 0.03 for trend on a continuous scale) in the fully adjusted logistic regression model. However, a recent Danish case-control study by Bellavia et al. found no significant association between diesel exhaust and ALS (OR = 0.79, 95% CI, 0.79–1.28) (58).

The case-control study by Visser et al. studied occupational exposure to other work-related air pollutants, particularly various particulates, through employment of job exposure matrices alongside face-to-face interviews and structured questionnaires (57). Occupational exposure to organic dust (*p* = 0.03) and silica (*p* = 0.01) were significantly associated to ALS on a continuous scale. However, only silica had a significant OR for both low (OR = 2.24; 95% CI, 1.50–3.35) and high (OR = 2.13; 95% CI, 1.42–3.20) exposures relative to those never exposed. Other occupational particulates investigated included animal contact and endotoxin, as well as some hazardous air pollutants (asbestos and polycyclic aromatic hydrocarbons), none of which were significantly associated with ALS on a continuous scale.

### 3.4. Qualitative synthesis of water pollution factors associated with the development of ALS

#### 3.4.1. Contaminated water and source of drinking water

Of the 3 studies that assessed exposure to high selenium content in water as a risk factor within a common study population, all of them found it to be positively associated with ALS. The residents of Reggio Emilia, Italy, were accidentally exposed to drinking water with high selenium content until 1988 with initial exposure starting potentially as early as 1972 (66). The exposed cohort was determined to be 5,182 residents based on a resident's address on the municipal water supply agency list of subscribers. During the follow-up until December 31, 1994, four cases of sALS were diagnosed, all occurring in cohort members with the longest ascertainable period of exposure. The SIR was 4.22 (95% CI, 1.15–10.80) in comparison to the reference group (remaining municipal population) with a higher SIR upon limiting the analysis to a sub-cohort of those with the longest ascertainable exposure period (SIR = 8.90, 95% CI = 2.43–22.79). In both analyses, a stronger effect was observed among females with a SIR of 9.77 (95% CI, 2.02–28.56) for the total cohort and of 21.36 (95% CI, 4.41–62.44) for the sub-cohort. A case-control study in 2010 by the same group on these residents exposed to drinking water with high selenium content reported that consumption of drinking water containing ≥1 μg/l of inorganic selenium was significantly associated with ALS (RR = 5.4, 95% CI = 1.1–26) even after adjustment for all potential confounders (educational attainment level and exposures to pesticides, industrial chemicals, and electromagnetic fields) (61). A combination of questionnaire-interviews, data from the local water agency and water samples were used for exposure ascertainment. A second follow-up period on these residents from 1995 to 2015 identified a crude incidence rate of ALS of 14 cases per 100,000 person-years in the exposed cohort as opposed to a crude incidence rate of ALS of 5 cases per 100,000 person-years in the unexposed cohort. Poisson regression analysis, adjusting for age, sex, and calendar year, revealed an overall incidence rate ratio (IRR) for ALS of 2.8 (95% CI, 1.3–6.0) with a substantially stronger IRR of 8.2 (95% CI, 1.8–14.3) in 1986–1994 than in 1995–2015 (IRR = 1.5, 95% CI, 0.5–4.7) (68). Stronger effects among women were noted once again with an IRR of 5.1 (95% CI, 1.8–14.3) as opposed to an IRR of 1.7 (95% CI, 0.5–5.4) in men. Despite the small quantities of studies, there is some strong evidence for high selenium content in drinking water as a risk factor for ALS. Studies in other regions would further strengthen the evidence if associations were found.

Outside of Reggio Emilia, another cohort study, a retrospective follow-up between 1979 and 2008, compared mortality rates between 154,932 marine and naval personnel exposed to drinking water contaminated by solvents (Camp Lejeune) and 154,969 marine and naval personnel unexposed to contaminated drinking water (Camp Pendleton) (67). Water samples were taken to assist with exposure ascertainment. Ultimately, fully adjusted comparisons of ALS mortality, 10-year lag, between the two cohorts did not reveal an elevated hazard ratio for ALS (0.83, 95% CI, 0.47–1.48).

There was one other paper that studied contaminated water (71). The study was prompted by a higher incidence of sALS in the Briga area in the province of Novara, Italy, between 2002 and 2012 in comparison with the entire province (72). In the Novara province, there were 106 ALS cases identified with an incidence rate of 4.65 per 100,000 for the Briga area. The study region was characterized as having severe environmental heavy metal pollution. Environmental data and water samples were thus taken for exposure ascertainment of the current included study (71). Results of a monitoring campaign carried out between 1989 and 1995 showed high metal contamination [Cr[III], Cr[IV], Cu, Ni, and Zn] in surface waters with severe metal contamination [Cr[III], Cu, Ni, and Zn] found at points sources (identifiable sources of pollution, such as industrial plants) and discharging into the fresh surface waters of the Briga area. Site-specific groundwater metal contamination (Al, Cu, Mn, and Ni) was also found in the Briga area despite diffuse contamination of groundwater in the Briga area was not demonstrated. These results, alongside the studies on drinking water containing high selenium content, all support the hypothesis of heavy metal contamination in water as a risk factor for ALS.

Beyond contaminated water, there were three papers identified that studied source of drinking water as a risk factor for ALS (35, 63, 65) with only one study indicating that those chronically exposed to raw water (untreated) had significantly increased odds of ALS in the fully adjusted model (OR = 4.74, 95% CI, 1.33–16.85) (65). Well water was not associated with ALS in all three studies, and this for both current use or ever use of a private well/fountain for drinking (63). Tube well, ponds and corporation supplied water were also all sources of drinking not associated with ALS (35). While these studies were of moderate quality, it should be noted that all three used either an open-ended structural interview (35) or self-administered questionnaire (63), or both (65) for exposure ascertainment with no water samples or environmental data used to evaluate well water quality among other sources of water.

#### 3.4.2. Proximity to water bodies and lakes prone to cyanobacterial blooms

Of the three studies that assessed proximity to water bodies as a risk factor, two found it to be positively associated with ALS. A case-control study in the New England region of the United States of America by Andrew et al. indicated that residing full-time within 2 miles of a water body (OR = 1.59; 95% CI, 1.05–2.42) is positively related to ALS status (62). However, some bias was possible with 90% of ALS cases having completed the questionnaire used for ascertainment of exposures, while only 52% of controls completed the questionnaire. A case-control study in Italy that used a questionnaire for exposure ascertainment also reported having ever lived <3 km from water bodies was significantly associated with the odds of ALS (OR = 1.83, 95% CI, 1.04–3.21) in adjusted models (41). However, a second case-control study in Italy utilizing geospatial data and of high quality reported no significant association between odds of ALS related to water bodies within 100 m from a subject's residence at the time of diagnosis or from the subject's residence in the former 30 years (64). Analyses by water body type (ex. lakes, rivers, reservoirs) were not conducted in these three included studies. Results are thus mixed.

Three studies all within the Northern New England region identified a potential link between lakes prone to algae blooms and ALS (24, 69, 70). A study by Caller et al. of sALS patients diagnosed between 1990 and 2007 in Enfield, New Hampshire, a town which encompasses a lake with a history of cyanobacteria algal blooms, identified nine ALS patients (10–25 times the expected annual incidence of 2 per 100,000) who lived within 0.5 miles of Lake Mascoma (69). Using a geocoded spatial database of ALS patients across New Hampshire, Vermont, and Maine, Torbick et al. revealed that 709 of the 754 cases had at least one lake within 10 km, while all cases had at least one lake within 30 km (24). There were several distinct ALS hotspots with decreasing secchi depth within a 30 km radius (OR = 0.40; 95% CI, 0.249–0.614) and increasing average total nitrogen within a 30 km radius (OR = 2.42; 95% CI, 1.460–4.124), both water-quality indicators of harmful algae blooms that are significantly associated with an increased probability of belonging to one of these clusters of ALS cases. Follow-up work by the same group further supported these results with a 48% increase in ALS risk noted when average phycocyanin concentration, a proxy measure for reoccurring seasonal cyanobacterial harmful algal blooms, is 100 μg/L (70). However, these studies were ecological in nature, albeit with the inclusion of field measurements.

Collectively, water-quality indicators and lake conditions that promote harmful algal blooms seem to be associated with ALS in the Northern New England region and should be studied further in epidemiological study designs higher up on the evidence hierarchy, as well as in other regions with lakes prone to cyanobacterial blooms.

#### 3.4.3. Water sports

In addition to inquiring about waterbody proximity among residents of New England, the case-control study by Andrew et al. in the United States of America included questions pertaining to participation in water sports in their questionnaire (62). Although frequent participation (≥2x per month, for 1 year or more) in water-skiing (OR = 3.89, 95% CI, 1.97–8.44) and boating, sailing, or kayaking (OR = 1.51, 95% CI, 1.01–2.28) were associated with an increased odds of ALS in crude models, only water-skiing remained significantly associated with ALS in adjusted models (adjusted OR = 3.71, 95% CI, 1.80–8.47). Swimming in lakes or rivers was not associated with ALS (OR = 1.43, 95% CI, 0.97–2.1). As indicated earlier, some bias may be possible given the large difference in response rate between ALS cases and controls.

## 4. Discussion

This series of three systematic reviews identified 44 epidemiological studies assessing relationships between urbanization, air pollution and water pollution with the development of ALS. Many potential environmental risk factors were identified that are consistently associated with a higher risk of ALS, capturing the ever-growing literature on environmental factors since previous systematic reviews. This is the first study to systematically synthesize all of this for three different types of environmental exposures (urbanization, air pollution and water pollution) and expands on previous systematic reviews that pertained to rural environments (25) or various individual environmental risk factors (e.g., heavy metals and other solvents) (15). It is also one of few reviews pertaining to ALS environmental factors to conduct qualitative appraisal of individual articles using criteria from a risk of bias assessment scale (73) and/or previously developed criteria (15). Potential risk factors, particularly regarding air and water pollution, were identified for each type of environmental exposure and are further discussed below.

### 4.1. Urbanization

While few studies found an association between rural areas and ALS (35, 43, 44, 46), others found higher risk in more urbanized areas (36, 50, 53). However, by large, most of the included studies found no association between place of residence and the risk of ALS when using designations of rural (32–34, 45, 47) or urban areas (34, 35, 43–45, 47, 48), or when using cut-offs for urbanization degree based on varying measures of population density (21, 49, 54). Scott et al. who reported population density alone explains 25% of the variance in adjusted relative risk for ALS, also found that the relationship between incidence and population density wasn't linear (50); thus, suggesting that there may be some other factor sometimes associated with residence rather than rural or urban residence itself being the causal link. As noted by the authors, the definition of an urban or rural area will differ across studies, as well as their environmental factors in the surrounding area depending on the country. Agricultural land itself did not appear to be linked with only one study out of four finding an association between agricultural areas and the risk of ALS (39). Among the four studies reporting a positive association with rural areas, over-representation of elementary occupations (including farmers) (43), farmers (46), or history of pesticide exposure (35, 44) all emerged as having potential influences. Thus, there may be a potential role of exposure to agricultural chemicals or animals linked to the agricultural work being the underlying influence of rural areas on disease risk (21). The case-control study by Das et al. that noted an association to history of pesticide exposure did not report one between ALS and farming (35), further proposing that agricultural chemicals, such as pesticides, may be one underlying risk factor responsible for such associations found between ALS and rural residence. This hypothesis is supported by the designation of pesticides as a probable risk factor according to the grading of evidence for exogenous ALS risk factors by Oskarsson et al. (16), as well as further supported by a systematic review on exposure to rural environments showing that the risk of ALS is significantly increased with pesticide exposure and with farmers (25). In fact, a recent study on geospatially estimated exposure to crop-applied pesticides for residence at diagnosis of ~26,000 nationally distributed ALS patients, and matched non-ALS controls, indicated potential neurotoxic pesticide exposures as risk factors for sporadic ALS (74). Among the statistically significant associations with evidence of neurotoxicity in the literature was glyphosate, the most widely used biocide on the planet (75). However, how these pesticides exhibit their toxicity mechanism is still being studied, varying from mechanisms of action involving inflammatory changes to interfering with nerve signal transmission (76–80). Various systematic reviews and/or meta-analyses with pesticides as an environmental risk factor of interest exist and should be consulted for those seeking further information (15, 25, 81–83). In addition to pesticides, agricultural practices often utilize heavy equipment powered by diesel engines. As for highly urbanized areas, air pollution levels could be a potential confounding factor associated to degree of urbanization (36, 84). Thus, diesel exhaust, which appears to be a risk factor for ALSS among the included studies, may be another important underlying factor to consider for the role of urbanization in the development of ALS. Studies mapping local climate zones, based on form and function of cities (e.g., residential, heavy industry, and scattered trees), may provide further progress toward understanding if environmental factors related to place of residence have a role in the development of ALS (17).

Exposure to electromagnetic fields and high-voltage infrastructure in the surrounding area showed an increased to the risk of ALS in three of five studies (41, 42, 51), however, larger case control studies with geospatial data are warranted given that one of these positive studies used an ecological approach (51), while the positive case-control study used a self-administered questionnaire for exposure assessment on a sample size below 100. There was also no association between ALS and electromagnetic fields/powerlines found in the larger case-control study by Vinceti et al. that utilized geospatial data (37). Thus, evidence is rather mixed for electromagnetic fields among the included studies. A recent meta-analysis by Filippini et al. also found scant evidence for a positive association between residential magnetic fields exposure and ALS (73). Eligibility criteria for study inclusion varied regarding how cases of ALS were identified and thus some of the excluded studies from this review were included in their meta-analysis (85–88). The authors noted very imprecise estimates in the studies and that the available data is still too limited, particularly for modeled magnetic field estimates (73). Ultimately, further studies are needed before very high exposure subgroup analyses can be conducted to rule out such associations between electromagnetic fields and the risk of ALS (73). Regarding biological plausibility, pathophysiological mechanisms for electromagnetic fields are far from concrete with oxidative stress modulation being a proposed hypothesis (89) given the role increased oxidative damage may play in neurological conditions such as ALS (90) and evidence that metabolic processes generating oxidants can be influenced by electromagnetic fields (91). Further investigation of factors associated to electrical occupations, beyond electromagnetic fields in the surrounding environment, may be warranted to Vergara et al. (92).

As for the various industrial related activities studied in a limited number of included articles with mixed results, future research should aim to use geospatial data as opposed to self-administered questionnaires in case-control study designs when databases are available, such as the geodata and maps used in the various electromagnetic field studies (37, 42, 51). Measurements on amount of pollutants emitted from such industrial facilities into the surrounding environment, directly or indirectly, would also be useful; particularly in areas with higher incidences of ALS (71, 72). Such studies would allow for a better understanding of how pollutants from these industrial facilities are interacting with the environment (ex. industrial wastewater discharges into rivers) and ultimately the municipal populations that surround them (18, 19).

### 4.2. Air pollution

Quite a few high-quality studies identified a positive association between residential exposure to atmospheric pollutants and the risk of ALS. Particularly, NO_2_ was significantly associated with increased risk of ALS in all three papers that studied this pollutant (36, 39, 60). A dose-response between NO_2_ and the odds of ALS was also noted which further points to the possibility of it being a risk factor for ALS (39). Exposure to air pollutants have also been recently investigated as a risk factor for Parkinson's disease (PD) (93). The retrospective cohort study revealed a significantly increased hazard ratio for PD in those exposed to NO_2_, but not in any of the other air pollution exposures (PM_2.5_, PM_10_, SO_2_, O_3_, and CO). Among the included studies pertaining to diesel exhaust exposure, three found a significant positive association with ALS (23, 55, 57), with significant overall trends for 5- and 10-year exposure lags suggesting the influence of diesel exhaust on ALS risk may be relevant many years prior to clinical onset (23). Typical diesel exhaust composition includes particulate matter, SO_2_ and NO_2_, among other components (94), further suggesting NO_2_ may play a role in the development of ALS. Biological plausibility for an association between air pollutants and neurodegeneration exists with fine particles from industrial activities and combustion inducing systematic inflammation when inhaled and lead to disruption of the blood-brain barrier's integrity (20). Additional lab studies have even noted potential suitable biomarkers for neurotoxicity due to exposure to air pollutants, such as particulate matter, with various immune responses occurring as microglia respond to different forms of outdoor ambient air pollution (95, 96).

While bi-pollutant models were run in a couple of the included studies (36, 60), interactions between pollutants that create deadly bi-products should be investigated as well, such as the creation of O_3_ from nitrogen oxides and volatile organic compounds in the atmosphere interacting with solar ultraviolet radiation (97). Exposure to SO_2_ was only investigated in one study (39) and should also be further investigated as it is a component of diesel exhaust (94) and has been shown to have a synergy effect on neurodegeneration mechanisms upon interaction with PM_2.5_ (98), which also had some suggestion for an association with ALS (36, 60). Studying such interactions may help to further explain the causal pathways of these air pollutants in the development of ALS.

### 4.3. Water pollution

The residents of Reggio Emilia, Italy, that were accidentally exposed to drinking water with high selenium content provided an epidemiological opportunity to follow-up on a large exposed cohort (61, 66, 68) and see if the rare outcome of ALS occurs. Ultimately, there was strong evidence for this heavy metal as a risk factor for ALS, particularly in the female residents of the area. While there were no included studies outside this region to support such associations to selenium, there have been case reports in South Dakota over a 10-year period of four patients living within a 15-km radius of each other in a region where naturally occurring selenium is of sufficient quantity in the soil to produce intoxication in livestock (99). Some heavy metals are trace elements and thus small amounts are essential to maintain various biological processes and metabolic functions of the body (100). However, in excess states, selenium may cause symptoms of central nervous system (CNS) disorder through numerous potential neurotoxic effects (101), including inhibition of glutamate uptake according to laboratory studies (102–104).

As for other heavy metals in contaminated water sources, the Briga area in the province of Novara, Italy, which was characterized as having increased incidence of sporadic ALS (72), found high contamination of chromium, copper, nickel, and zinc in surface waters and fresh surface waters, as well as site-specific groundwater metal contamination of aluminum, copper, manganese and nickel (71). Among them, manganese have been noted to have a potential role in neurodegenerative diseases, such as parkinsonian syndrome (105), and possibly acts as a toxicant *via* its pro-oxidant activity (106). However, like selenium, the role of such heavy metals in motor neuron degeneration may be better understood by finding common underlying mechanisms. Looking for their increased presence within the neurons of those people living with ALS (107), particularly in environments with high risk of exposure, as to see if uptake occurred may be beneficial as well, such as the low levels of calcium and zinc in the drinking water of the Kii Peninsula area of Japan resulting in a finding that patients with K area ALS (K-ALS) had low serum levels of these elements and an increase in oxidative stress markers (108, 109). Systematic reviews and/or meta-analyses with heavy metals as an exposure of interest (not limited to heavy metal contamination in water sources or *via* air pollution) exist and should be consulted for those seeking further information on their potential role in ALS etiology (15, 83).

Analyses of proximity to waterbodies by type (e.g., lakes, rivers, and reservoirs) were not conducted in the three included studies (41, 62, 64). Results were thus mixed. Differentiating by type of water feature may highlight associations given that living near waterbodies was used as a proxy of cyanobacteria exposure (62, 64). Low water flow conditions, such as in lakes, ponds, and reservoirs, or even rivers slowed by dams, is one of many factors that can promote harmful algae blooms and cyanobacterial growth (110, 111). Nutrients, such as phosphorus and nitrogen, can greatly contribute to eutrophication and support cyanobacterial growth with fertilizers or even discharged wastewater being potential sources (111, 112). While the three included studies finding an association between cyanobacterial prone lakes and ALS cases or hotspot membership were all ecological in nature (24, 69, 70), they did include some field measurements that highlighted the presence of water quality indicators that promote harmful algal blooms. However, proximity does not necessarily mean causation. As for pathological mechanisms, the neurotoxin β-methylamino-L-alanine (BMAA), a compound that can be produced by cyanobacteria (113), has been the proposed molecular culprit (114, 115). Supporting evidence includes the endemic cases of amyotrophic lateral sclerosis-parkinsonism-dementia (ALS-PDC) on the island of Guam in which BMAA was detected in brain proteins of those affected by PD, Alzheimer's disease, and ALS, but absent in controls (116), with chronic exposure through dietary consumption (117). Misincorporation of BMAA into ALS-linked proteins has been reported (116, 118), however, the mechanisms responsible for BMAA toxicity in neurodegeneration are still being investigated with a causal link yet to be found outside of Guam in relation to progressive neurodegenerative diseases (119). Associations described herein should be further studied with better epidemiological study designs and measures, as well as in regions outside New England.

For water sports, only frequent participation in water-skiing was significantly associated with ALS in the final adjusted models (62). The study was conducted in the New England region, where other studies pertaining to cyanobacterial blooms have been done (24, 69, 70), among residents. As noted by the authors, inhalation of aerosolized particles of cyanotoxins as an exposure route has been previously proposed (120) and could be generated in activities such as waterskiing on bodies of water with cyanobacteria present. However, more studies on watersport participation are needed and in other areas with lakes prone to cyanobacterial blooms to support the association found in this single case-control study.

### 4.4. ALS: A complex multifactorial neurodegenerative disease

The hypothesis in the field of ALS that the disease is a complex genetic condition means that contributions from multiple genetic and environmental risk factors are likely involved in the development of ALS, with relative effect size from individual factors being rather small (12). Environmental factors may have complex relations between one another in their correlational or causal relationship with disease that extends beyond additive models (121), such as pollutants emitted from nearby industrial activities reacting with other pollutants in a synergy manner (98) or even a multifactorial manner upon interacting with their surrounding environment (e.g., acidification and eutrophication) (112). Influence of such environmental conditions on disease development may even be moderated by genetic predisposition (122). The low prevalence of the disease and likely latent response of environmental risks with time acting on pre-existing genetic load also makes studying such exogenous factors mostly unfeasible with prospective cohort studies and therefore requires the use of case-control studies using historical measures of exposures that avoid recall bias (8, 123, 124). Large datasets including both genetic testing results in addition to geospatial data on environmental risk factors would potentially allow for identification of such environmental triggers in distinct sub-populations and/or in a temporal manner (12, 125). While several of the included case-control studies had geospatial information which allowed taking consideration of historical exposures in the surrounding environment of individuals, particularly among environmental factors related to air pollution, few studies utilized lag models (23, 58, 67) and many urbanization and water pollution studies relied merely on residence at onset for ascertaining associations to ALS. Additional studies with longitudinal high-resolution geospatial measurements and residential histories of participants are recommended to strengthen confidence in associations documented and potentially identify new underlying risk factors in the surrounding environment of those who develop ALS (126).

The use of a combination of keywords/MeSH terminology to identify records was a strength for this series of systematic reviews (26). The study-level risk of bias assessment for studies included was another strength (37, 41) and it concorded with the qualitative assessments from other reviews (73) despite using a different risk of bias instrument (127). However, it should be noted that a systematic review itself cannot be used to determine the causality of a risk factor in the development of ALS. Limitations include it is possible that the list of risk factors investigated was not complete. Gray literature was not screened and the various filters utilized only allowed identification of research articles in French or English. The absence of studies pertaining to pesticides or heavy metals such as lead, both of which are probable risk factors according to Oskarsson et al. (16), is due to their exposure not being associated to air or water pollution in the surrounding environment (15, 25, 81–83). Inclusion of literature indirectly related to the three main exposures of interest could be an avenue to explore for future reviews, such as studies on consumption of fish from lakes (128, 129). Finally, only environmental studies and findings were investigated and appraised, whereas reviews synthesizing both genetic and environmental studies may highlight important gene-environment interactions based on the existing evidence (15).

## 5. Conclusions

The current series of three systematic reviews sought to appraise the growing literature of environmental factors pertaining to urbanization, air pollution and water pollution in the development of ALS. Studies on environmental factors pertaining to urbanization of the surrounding area were the most studied in the context of this series of systematic reviews but presented mixed results for an association to ALS. The relationship of air pollutants to ALS was clearer with results suggesting that diesel exhaust exposure and nitrogen dioxide, a primary product of diesel combustion, are linked to an increased risk of ALS. Heavy metal contamination in water, particular selenium, as well as proximity of residence to lakes prone to cyanobacterial blooms, was associated with an increased risk of ALS. Measures to reduce air and water pollution are important as diesel exhaust, nitrogen dioxide and poor water quality all appear to represent modifiable risk factors of ALS.

## Data availability statement

The original contributions presented in the study are included in the article/Supplementary material, further inquiries can be directed to the corresponding author.

## Author contributions

DS conceived the current study, performed the literature search, appraised the included articles, extracted the pertinent data, and drafted the initial manuscript. PPWR independently appraised and extracted data from a subsample of the included articles. MB resolved any disagreements between DS and PPWR. DS, MB, and CO'C revised the intellectual content of the work critically. All authors approved the final version to be published.
