# Supplementary material for: Urbanization, air pollution, and water pollution: Identification of potential environmental risk factors associated with amyotrophic lateral sclerosis using systematic reviews
Source: Front Neurol. 2023 Mar 8;14:1108383. doi: 10.3389/fneur.2023.1108383 (PMC10030603; doi:10.3389/fneur.2023.1108383)
Supplement: Supplementary file 1 [file Data_Sheet_1.docx]

Supplementary Material

**Supplementary Materials Content**

**Supplementary Methods 1.** Search strategy used in the current systematic reviews (n = 3).

**Supplementary Methods 2.** Newcastle-Ottawa quality assessment scale guide for case control studies.

**Supplementary Methods 3.** Newcastle-Ottawa quality assessment scale guide for cohort studies.

**Supplementary Methods 4.** Modified Newcastle-Ottawa risk of bias scoring guide for cross-sectional studies.

**Table S1.** Summary of study characteristics (region, method of exposure ascertainment, study design and Newcastle-Ottawa Scale quality assessment) for each exposure of interest (n = 3).

**Table S2.** Supplementary description (ALS diagnostic criteria and source of cases and controls) of 25 included urbanization studies

**Table S3.** Supplementary description (ALS diagnostic criteria and source of cases and controls) of 9 included air pollution studies

**Table S4.** Supplementary description (ALS diagnostic criteria and source of cases/exposed and controls/unexposed) of 14 included water pollution studies

**Table S5.** Newcastle-Ottawa quality assessment scores for the 24 case-control studies included in the conducted systematic reviews (n = 3)

**Table S6.** Newcastle-Ottawa risk quality assessment scores for the 3 cohort studies included in the conducted systematic reviews (n = 3)

**Table S7.** Modified Newcastle-Ottawa risk of bias scores for the 2 cross-sectional studies included in the conducted systematic reviews (n = 3)

This supplementary material has been provided by the authors to give readers additional information about their work.

**Supplementary Methods 1.** Search strategy used in the current systematic reviews (n = 3). (Pages 53 – 56)

**PubMed**

Amyotrophic lateral sclerosis

1. Amyotrophic lateral sclerosis
2. ALS
3. Motor neuron disease
4. Motor neuron diseases
5. Motor neurone disease
6. Motor neurone diseases
7. MND
8. Lou Gehrig’s disease
9. Lou-Gehrig’s disease
10. Lou Gehrigs disease
11. Lou-Gehrigs disease
12. Gehrig’s disease
13. Gehrigs disease
14. Gehrig disease
15. Lou-Gehrig disease
16. Lou Gehrig disease
17. Charcot disease
18. Charcot’s disease
19. Motor neuropathies
20. Motor neuropathy
21. Progressive muscular atrophy
22. Progressive spinal muscular atrophy
23. Primary lateral sclerosis
24. Progressive bulbar atrophy
25. Spinal muscular atrophy
26. Amyotrophic Lateral Sclerosis [MeSH]
27. OR / 1-26

Urbanization

1. Rural
2. Urban
3. Urbanization
4. Residential
5. Commercial
6. Industrial
7. Trees
8. Tree
9. Forest
10. Grass
11. Vegetation
12. Green space
13. Green area
14. Neighbourhood
15. Housing
16. Houses
17. House
18. Condos
19. Condo
20. High-rises
21. High-rise
22. Paved
23. Water features
24. Water feature
25. Climate zone
26. Population density
27. Addresses
28. OR / 28-54

Air pollution

1. Air pollution
2. Air pollutions
3. Air quality
4. Air pollutants
5. Particulate matter
6. Traffic pollution
7. Vehicle pollution
8. Traffic-related pollution
9. Traffic related pollution
10. Vehicle emissions
11. Volatile organic compounds
12. VOCs
13. Air Pollution [MeSH]
14. Traffic-Related Pollution [MeSH]
15. OR / 56-69

Water pollution

1. Water quality
2. Water pollution
3. Water pollutions
4. Water pollutants
5. Waster water
6. Lakes
7. Lake
8. Rivers
9. River
10. Seas
11. Sea
12. Groundwater
13. Drinking water
14. Oceans
15. Ocean
16. Well water
17. Algae
18. OR / 71-87

Observational studies

1. Epidemiologic Studies [MeSH]
2. Cohort Studies [MeSH]
3. Cohort
4. Longitudinal
5. Prospective
6. Retrospective
7. Case-control
8. Cross-sectional
9. OR / 89-96

Filters

1. Humans
2. English
3. French

Combined searches (n = 3)

Urbanization: (((#27) AND (#55)) AND ((#97))) AND ((#98) AND (#99 OR #100))

Results: 652 records

Air pollution: (((#27) AND (#70)) AND ((#97))) AND ((#98) AND (#99 OR #100))

Results: 33 records

Water pollution: (((#27) AND (#88)) AND ((#97))) AND ((#98) AND (#99 OR #100))

Results 150 records

For all three combined searches, indexed articles since database inception were searched on March 24, 2020, with an update to these three initial searches conducted on October 25, 2021, to cover the period of March 25, 2020, to October 25, 2021.

**Abbreviations:** MeSH, Medical Subject Heading in PubMed

**Scopus**

A systematic mining of the various types of citation relationships between articles was done conducted as described by Belter (2016) ([1](#_ENREF_1)) to perform citation analysis within Scopus. One to two known articles pertaining to each exposure of interest (Urbanization ([2](#_ENREF_2), [3](#_ENREF_3)); air pollution ([4](#_ENREF_4), [5](#_ENREF_5)); water pollution ([6](#_ENREF_6))) were used as the original key articles from which direct citations (cited papers and citing papers) and indirect citations (co-citing papers and co-cited papers) were retrieved.

For all three citation analyses, indexed citations since database inception were searched on March 24, 2020, with an update to these three initial analyses conducted on October 25, 2021, to cover the period of March 25, 2020, to October 25, 2021. Below are three flow of information charts (one for each exposure of interest) showing the number of unknown records identified through citation analysis on Scopus of the known key papers via the four types of citation relationships between papers. Note that co-citing records and co-cited records were refined and filtered within Scopus by using search terms and applying filters (document type [article] and language [English and French]) prior to extraction to Mendeley due to the vast quantity of identified records via these indirect citation relationships.

Urbanization (802 unique records identified)


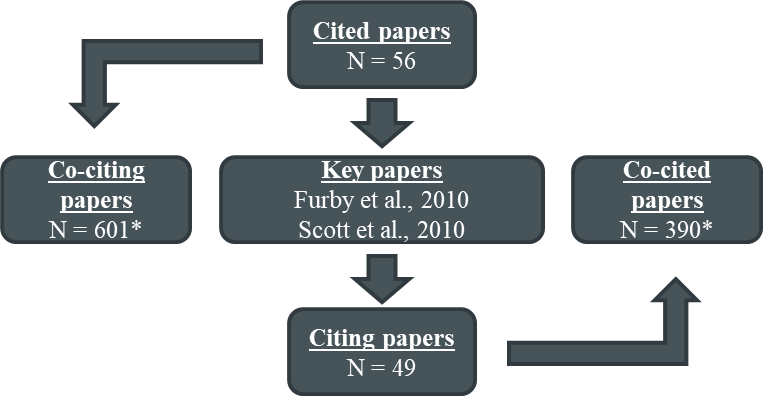


* Urbanization co-citing and co-cited search terms and applied filters: ("amyotrophic lateral sclerosis" OR "motor neuron disease" OR "Lou Gehrig's Disease" OR "Charcot Disease" OR "primary lateral sclerosis" OR "spinal muscular atrophy" OR "progressive muscular atrophy" AND rural OR urban OR urbanization OR industrial OR tree) AND (LIMIT-TO (DOCTYPE, "ar")) AND (LIMIT-TO (LANGUAGE, "English") OR LIMIT-TO (LANGUAGE, "French"))

Air pollution (413 unique records identified)


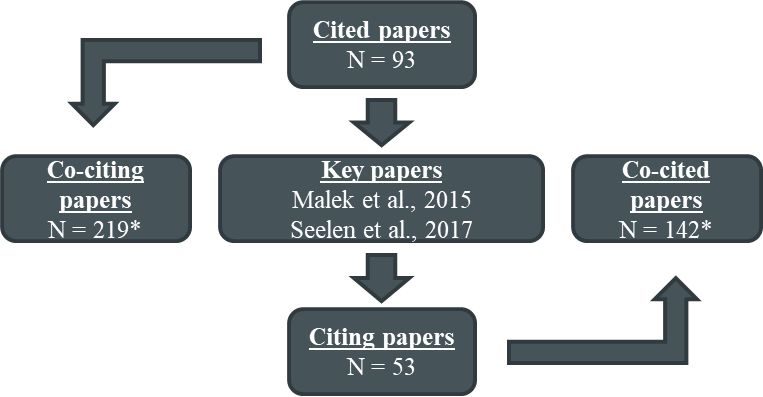


* Air pollution co-citing and co-cited search terms and applied filters: ("amyotrophic lateral sclerosis" OR "motor neuron disease" OR "Lou Gehrig's Disease" OR "Charcot Disease" OR "primary lateral sclerosis" OR "spinal muscular atrophy" OR "progressive muscular atrophy" AND air OR "volatile organic matter" OR traffic) AND (LIMIT-TO (DOCTYPE, "ar")) AND (LIMIT-TO (LANGUAGE, "English") OR LIMIT-TO (LANGUAGE, "French"))

Water pollution (1087 unique records identified)


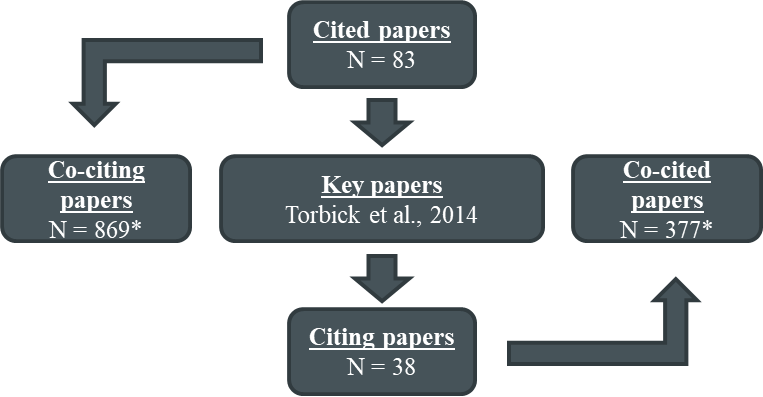


* Water pollution co-citing and co-cited search terms and applied filters: ("amyotrophic lateral sclerosis" OR "motor neuron disease" OR "Lou Gehrig's Disease" OR "Charcot Disease" OR "primary lateral sclerosis" AND water OR lake OR river OR ocean OR sea OR groundwater OR “well water” OR “waste water” OR “drinking water” OR algae) AND (LIMIT-TO (DOCTYPE, "ar")) AND (LIMIT-TO (LANGUAGE, "English") OR LIMIT-TO (LANGUAGE, "French"))

**Supplementary Methods 2.** Newcastle-Ottawa quality assessment scale guide for case control studies.

Note: A study can be awarded a maximum of one star for each numbered item within the Selection and Exposure categories. A maximum of two stars can be given for Comparability.

**Selection**

1) Is the case definition adequate?

a) yes, with independent validation (ex. diagnostic criteria, electromyography, neurologist) *

b) yes, via record linkage only (ex. identified through ICD codes on database records)

c) no description

2) Representativeness of the cases

a) consecutive or obviously representative series of cases *

b) potential for selection biases or not stated

3) Selection of Controls

a) community controls *

b) hospital controls

c) no description

4) Definition of Controls

a) no history of disease (endpoint) *

b) no description of source

**Comparability**

1) Comparability of cases and controls on the basis of the design or analysis

a) study controls for age *

b) study controls for gender/sex *

**Exposure**

1) Ascertainment of exposure

a) secure record (ex. field measurements, government databases, remote sensing) *

b) structured interview where blind to case/control status *

c) interview not blinded to case/control status

d) written self report or medical record only

e) no description

2) Same method of ascertainment for cases and controls

a) yes *

b) no

3) Non-Response rate (or missing data rate)

a) same rate for both groups *

b) rate different and non respondents described

c) rate different and/or no designation

**Supplementary Methods 3.** Newcastle-Ottawa quality assessment scale guide for cohort studies.

Note: A study can be awarded a maximum of one star for each numbered item within the Selection and Outcome categories. A maximum of two stars can be given for Comparability

**Selection**

1) Representativeness of the exposed cohort

a) truly or somewhat representative of the average (exposed) in the community *

c) sampled from a special population (ex. company, hospital patients, insurance data)

d) no description of the derivation of the cohort

2) Selection of the non exposed cohort

a) drawn from the same community as the exposed cohort *

b) drawn from a different source

c) no description of the derivation of the non exposed cohort

3) Ascertainment of exposure

a) secure record (ex. field measurements, government databases, remote sensing) *

b) structured interview *

c) written self report

d) no description

4) Demonstration that outcome of interest was not present at start of study

a) yes *

b) no

**Comparability**

1) Comparability of cohorts on the basis of the design or analysis

a) study controls for age *

b) study controls for gender/sex *

**Outcome**

1) Assessment of outcome

a) independent or blind assessment or by reference to secure records (ex. medical files) *

b) record linkage (ex. identified through ICD codes on database records) *

c) self report (no reference to original medical records)

d) no description

2) Was follow-up long enough for outcomes to occur

a) yes (5+ years) *

b) no

3) Adequacy of follow up of cohorts

a) complete follow up - all subjects accounted for *

b) subjects lost to follow up unlikely to introduce bias (>= 80% follow up rate or description provided of those lost to follow up) *

c) follow up rate <80% and no description of those lost, or simply no statement

**Supplementary Methods 4.** Modified Newcastle-Ottawa risk of bias scoring guide for cross-sectional studies.

Note: The individual components listed below are summed to generate a total Modified Newcastle-Ottawa risk of bias score for each study. Total scores range from 0 to 5.

**(1) Sample representativeness:**

1 point: Sample is likely representative of ALS cases in the study’s target region with cases included from multiple hospitals, the region’s main ALS/MND clinic, an ALS registry, or a combination of multiple other sources (ex. death certificates, drug prescription directories, private neurologists).

0 points: Sample is likely not representative of ALS cases in the study’s target region with cases only included from a single hospital (other than main ALS/MND clinic) or other source (ex. death certificates, drug prescription directories or private neurologists).

**(2) Sample size:**

1 point: Sample size was greater than or equal to 200 participants.

0 points: Sample size was less than 200 participants.

**(3) Non-respondents:**

1 point: Comparability between respondent and non-respondent characteristics

was established with a satisfactory response rate (>= 80%). For studies utilizing medical records only, missing data resulted in exclusion of <10% of the finally qualified cases.

0 points: The comparability between respondents and non-respondents was

unsatisfactory, the response rate was unsatisfactory (<80%), or there was no description of the response rate or the characteristics of the responders or non-responders. For studies utilizing medical records only, missing data resulted in exclusion of>=10% of the finally qualified cases.

**(4) Ascertainment of exposure:**

1 point: The study employed the use of a secure record (ex. field measurements, government databases, remote sensing) or a structured interview (use of validated questionnaire or a set of standardized and premediated questions).

0 points: The study employed an unstructured interview (did not rely on valid questionnaire or set of premeditated questions), used self report or medical records only or did not provide a description for method of ascertainment of exposure.

**(5) Quality of descriptive statistics reporting:**

1 point: The study reported descriptive statistics to describe the population (ex. age, sex, clinical features) with proper measures of dispersion (ex. mean, standard deviation).

0 points: The study did not report descriptive statistics, incompletely reported

descriptive statistics or did not report measures of dispersion.

**Table S1.** Summary of study characteristics (region, method of exposure ascertainment, study design and Newcastle-Ottawa Scale quality assessment) for each exposure of interest (n = 3).

| **Systematic review**  **(*n* studies)** | **Region**  **(*n* studies)** | **Method of exposure ascertainment**  **(*n* studies)** | **Study design**  **(*n* studies)** | **NOS qualitative assessment ^a^**  **(*n* studies)** |
| --- | --- | --- | --- | --- |
| Urbanization (25) | Italy (10^b^)  USA (4)  France (3)  Ireland (3^b^)  Argentina (1)  China (1)  Greece (1)  Netherlands (2^b^)  Spain (1)  UK (1) | Medical Records (12)  Census data (9)  Questionnaire (6)  Geospatial data (5)  Interview (5)  Job-exposure matrix (1)  Other (1) | Matched case-control (10)  Nested matched case-control (1)  Pooled matched case-control (1)  Natural History [Cohort] (1)  Cross-sectional (2)  Ecological (10) | *NOS*  Moderate quality (6)  High quality (7)  *Modified NOS*  Low risk of bias (2)  N/A (10) |
| Air pollution (9) | Denmark (2)  Netherlands (3^b^)  Australia (1)  Italy (2^b^)  Ireland (1^b^)  Spain (1)  USA (1) | Geospatial data (5)  Medical Records (4)  Job-exposure matrix (3)  Questionnaire (3)  Other (2)  Census (1)  Interview (1) | Matched case-control (5)  Case-control (2)  Nested matched case-control (2) | *NOS*  High quality (8)  Moderate quality (1) |
| Water pollution (14) | Italy (9)  USA (5) | Questionnaire/survey (8)  Field measurements (5)  Geospatial data (4)  Interview (4)  Other (4)  Census (1)  Medical Records (1) | Matched case-control (6)  Prospective cohort (2)  Retrospective cohort (1)  Case-control (1)  Ecological (4) | *NOS*  Moderate quality (6)  High quality (4)  N/A (4) |

Abbreviations: N/A, not applicable; NOS, Newcastle-Ottawa Scale; UK, United Kingdom; USA, United States of America

^a^ Score and classification of study’s quality according to their assessment as described in Section 2.3.

^b^ Includes a pooled European study that included participants from Italy, Ireland, and the Netherlands.

**Table S2.** Supplementary description (ALS diagnostic criteria and source of cases and controls) of 25 included urbanization studies

| Author, year, [Reference] | ALS diagnostic criteria | Source of cases/exposed | Source of controls/unexposed |
| --- | --- | --- | --- |
| Case-control | | | |
| Armon et al., 1991 ([7](#_ENREF_7)) | Upper and lower motor neuron involvement, diagnosed by a neurologist and EMG studies confirming the diagnosis | Mayo Clinic | Accompanying persons, peer, or patient control |
| Cruz et al., 1999 ([8](#_ENREF_8)) | Diagnosed by at least one neurologist and their medical records subsequently reviewed by study neurologist to confirm; Patients exhibiting progressive MND of both upper and lower motor neurons, PBP, or PMA included | Referred by neurologists and two neurological disease support service organizations | Medicare enrollment lists (general population) |
| Muddasir Qureshi et al., 2006 ([9](#_ENREF_9)) | El Escorial criteria (suspected, possible, probable, or definite ALS) | Neuromuscular clinic | Accompanied the ALS subjects during clinic visits |
| Furby et al., 2010 ([2](#_ENREF_2)) | El Escorial criteria (probable or definite sALS) | Orthopedic service of hospital | Orthopedic service of hospital |
| Das et al., 2012 ([10](#_ENREF_10)) | Revised El Escorial criteria (definite) | Neurological referral center | Accompanying persons if available, and from those attending the neurology department |
| Yu et al., 2014 ([11](#_ENREF_11)) | Revised El Escorial criteria (possible, probable, probable lab-supported, or definite ALS) | ALS clinic | Recruited through postings and university website |
| Seelen et al., 2017 ([5](#_ENREF_5)) | Revised El Escorial criteria (possible, probable [laboratory supported], or definite) | Prospective ALS in the Netherlands (PAN) study | Registers of the patients’ general practitioners |
| Vinceti et al., 2017a ([12](#_ENREF_12)) | ICD-9 code 335.2 (MND) and then records reviewed by two neurologists to validate ALS diagnosis; Revised El-Escorial criteria (possible, probable, or definite) for ALS Registry | Death certificates; Emilia-Romagna Region ALS Registry (ERRALS); hospital discharge records; riluzole prescriptions; three databases from the National Health Service | National Health Services directories |
| Vinceti et al., 2017b ([13](#_ENREF_13)) | Revised El Escorial criteria (probable or definite) | ALS Emilia Romagna Registry (ERRALS); death certificates; drug prescription directories; hospital discharge records | National Health Service  directory |
| Povedano et al., 2018 ([14](#_ENREF_14)) | El Escorial criteria | Motor Disease Functional Unit | Subjects who had contact with the unit services |
| Peters et al., 2019 ([15](#_ENREF_15)) | Revised El Escorial criteria (possible, [laboratory-supported] probable, or definited) | Euro-MOTOR project | Euro-MOTOR project |
| Filippini et al., 2020a ([16](#_ENREF_16)) | Revised El Escorial criteria (probable or definite) | Recruited at hospitals or by mail via identification using death certificates; hospital discharge records; riluzole prescriptions; two ALS Registries (ERRALS and PARALS) | Recruited by mail via National Health Service directory of residents |
| Cohort | | | |
| Luna et al., 2019 ([17](#_ENREF_17)) | Airlie House criteria (possible, probable laboratory-supported, probable, or definite) | ALS French register in Limousin (FRALim) [non-classical cohort study] | Remaining population in region [non-classical cohort study] |
| Cross-sectional | | | |
| Bettini et al., 2011 ([18](#_ENREF_18)) | El Escorial (probable or definite sALS) | Motoneuron Disease Referral Centre | N/A |
| Wei et al., 2015 ([19](#_ENREF_19)) | Revised El Escorial criteria | Tertiary referral centre | N/A |
| Ecological | | | |
| Juvarra et al., 1983, ([20](#_ENREF_20)) | Clinical findings of first and second motor neuron involvement and/or labioglossopharyngeal muscles paralysis, in the absence of sensory disturbances, typical EMG findings, and absence of major spinal changes, confirmed by plain films and/or myelography | Clinical records of neurological and neurosurgical units of Parma (city and province) and orthopedic hospitals in areas without neurological facilities | N/A |
| Kalfakis et al., 1991 ([21](#_ENREF_21)) | Signs of lower and upper motor neuron involvement, no sensory or sphincter disturbances, positive electromyographic findings and no X-ray or biochemical findings indicative of other disease | University Department of Neurology | N/A |
| Bettoni et al., 1994 ([22](#_ENREF_22)) | Clinical findings of first and second motor neuron involvement and/or labioglossopharyngeal muscles paralysis, in the absence of sensory disturbances, normality of ocular movements and sphincters, typical EMG findings, and absence of major spinal changes, confirmed by myelography, CT scan and/or MRI | Clinical records of neurological and neurosurgical units of Parma (city and province) and geriatric and orthopedic hospitals in areas without neurological facilities | N/A |
| Mandrioli et al., 2003 ([23](#_ENREF_23)) | Revised El Escorial criteria | Clinical records of all hospitals and neurologic centers of province (ICD-9 code 335.2) | N/A |
| Govoni et al., 2005 ([24](#_ENREF_24)) | El Escorial (probable or definite) | Archives of the neurological ward of the University Hospital, of other neurological practices, of clinical neurophysiology services and of neurological out-patient services | N/A |
| Scott et al., 2010 ([3](#_ENREF_3)) | El Escorial criteria | Catchment area of the South-East England ALS Register (SEALS register) | N/A |
| Boumédiène et al., 2011 ([25](#_ENREF_25)) | El Escorial or Revised El Escorial criteria (probable or definite) | ALS center | N/A |
| Mandrioli et al., 2014 ([26](#_ENREF_26)) | Revised El Escorial criteria | Emilia Romagna Registry for ALS – ERRALS | N/A |
| Rooney et al., 2014 ([27](#_ENREF_27)) | El Escorial criteria | Irish ALS Register | N/A |
| Rooney et al., 2015 ([28](#_ENREF_28)) | El Escorial criteria | Irish ALS Register | N/A |

Abbreviations: ALS, amyotrophic lateral sclerosis; CT, computed tomography; EMG, electromyography; ICD, International Statistical Classification of Diseases and Related Health Problems; MND, motor neuron disease; MRI, magnetic resonance imaging; N/A, not applicable; PBP, progressive bulbar palsy; PMA, progressive muscular atrophy; sALS, sporadic amyotrophic lateral sclerosis

**Table S3.** Supplementary description (ALS diagnostic criteria and source of cases and controls) of 9 included air pollution studies

| Author, year, [Reference] | ALS diagnostic criteria | Source of cases | Source of controls |
| --- | --- | --- | --- |
| Case-control | | | |
| Pamphlett et Rikard-Bell, 2013 ([29](#_ENREF_29)) | Revised El Escorial criteria (probable or definite) | ALS Associations | ALS Associations |
| Malek et al., 2015 ([4](#_ENREF_4)) | El Escorial criteria (possible, probable, or definite) | Three neurology clinics with ALS centers | Mailing list; outpatient hospital controls from general neurological practice waiting rooms |
| Seelen et al., 2017 ([5](#_ENREF_5)) | Revised El Escorial criteria (possible, probable [laboratory supported], or definite) | Prospective ALS in the Netherlands (PAN) study | Registers of the patients’ general practitioners |
| Dickerson et al., 2018 ([30](#_ENREF_30)) | ICD-8 code 348.0 (ALS), and ICD-10 code G12.2 (MND) | Danish National Patient Registry | Danish Central Person Registry |
| Povedano et al., 2018 ([14](#_ENREF_14)) | El Escorial criteria | Motor Disease Functional Unit | Subjects who had contact with the unit services |
| Visser et al., 2019 ([31](#_ENREF_31)) | Revised El Escorial criteria (possible, probable, or definite [laboratory supported]) | Euro-MOTOR consortium | Euro-MOTOR consortium |
| Bellavia et al., 2021 ([32](#_ENREF_32)) | ICD-8 code 348.0 (ALS), and ICD-10 code G12.2 (MND) | Danish National Patient Registry | Danish Central Person Registry |
| Filippini et al., 2021 ([33](#_ENREF_33)) | Revised El Escorial criteria (probable or definite) | ALS Center | Individuals admitted to the same Neurological Department |
| Yu et al., 2021 ([34](#_ENREF_34)) | Revised El Escorial criteria (possible, probable, or definite) | Prospective ALS in the Netherlands (PAN) study | Registers of the patients’ general practitioners |

Abbreviations: ALS, amyotrophic lateral sclerosis; ICD, International Statistical Classification of Diseases and Related Health Problems; MND, motor neuron disease

**Table S4.** Supplementary description (ALS diagnostic criteria and source of cases/exposed and controls/unexposed) of 14 included water pollution studies

| Author, year, [Reference] | ALS diagnostic criteria | Source of cases/exposed | Source of controls/unexposed |
| --- | --- | --- | --- |
| Case-control | | | |
| Vinceti et al., 2010 ([35](#_ENREF_35)) | Revised El Escorial criteria (probable or definite) | Death certificates (ICD-9 code 335.2); prescriptions of riluzole; inpatients and outpatients from public and private hospitals (ICD-9 code 335.2) | Annual directories of residents |
| Das et al., 2012 ([10](#_ENREF_10)) | Revised El Escorial criteria (definite) | Neurological referral center | Accompanying persons if available, and from those attending the neurology department |
| Andrew et al., 2017 ([36](#_ENREF_36)) | Awaji-modified El Escorial criteria (probable or definite), including PMA diagnoses | ALS Center | Neurology clinic patients with other idiopathic diseases |
| Filippini et al., 2020a ([16](#_ENREF_16)) | Revised El Escorial criteria (probable or definite) | Recruited at hospitals or by mail via identification using death certificates; hospital discharge records; riluzole prescriptions; two ALS Registries (ERRALS and PARALS) | Recruited by mail via National Health Service directory of residents |
| Filippini et al., 2020b ([37](#_ENREF_37)) | Revised El Escorial criteria (probable or definite) | Recruited at hospitals or by mail/phone via identification using death certificates; hospital discharge records; riluzole prescriptions; two ALS Registries (ERRALS and PARALS) | Recruited by mail/phone via National Health Service directory of residents |
| Fiore et al., 2020 ([38](#_ENREF_38)) | Revised El Escorial criteria (probable or definite) | ALS Emilia-Romagna Registry (ERRALS); death certificates; drug prescription directory; hospital discharge directory | Local Health Authority Registries |
| Stipa et al., 2021 ([39](#_ENREF_39)) | El Escorial criteria (suspected, possible, probable, or definite) | Neurological Centre | Neurological Centre |
| Cohort | | | |
| Vinceti et al., 1996 ([40](#_ENREF_40)) | Neurologist using standard criteria | List of municipal water supply agency subscribers | Remaining municipal population |
| Bove et al., 2014 ([41](#_ENREF_41)) | National Death Index | U.S. Marine Corps Base Camp Lejeune | U.S. Marine Corps Base Camp Pendleton |
| Vinceti et al., 2019 ([42](#_ENREF_42)) | Record linkage | List of municipal water supply agency subscribers | Remaining municipal population |
| Ecological | | | |
| Caller et al., 2009 ([43](#_ENREF_43)) | Review of medical records | ALS Center; community databases | N/A |
| Torbick et al., 2014 ([6](#_ENREF_6)) | Review of medical records; Social Security Death Index | ALS Center; Muscular Dystrophy Association; surveys | N/A |
| Torbick et al., 2018 ([44](#_ENREF_44)) | Review of medical records; Social Security Death Index | ALS Center; Muscular Dystrophy Association; surveys | N/A |
| Tesauro et al., 2021 ([45](#_ENREF_45)) | ([46](#_ENREF_46))  El Escorial-revised classification (clinically probable, clinically probable-laboratory-supported, clinically definite) | ([46](#_ENREF_46))  Death certificates (ICD-9-CM code 335.20); hospital discharge records of ALS centre and a neurology department (ICD-9-CM code 335.20); Piemonte and Valle d’Aosta registry for ALS (PARALS) | N/A |

Abbreviations: ALS, amyotrophic lateral sclerosis; ICD, International Statistical Classification of Diseases and Related Health Problems; ICD-CM, International Statistical Classification of Diseases and Related Health Problems – Clinical Modification; N/A, not applicable; PMA, progressive muscular atrophy

**Table S5.** Newcastle-Ottawa quality assessment scores for the 24 case-control studies included in the conducted systematic reviews (n = 3)

| Reference | Selection | | | | Comparability | Exposure | | | Total |
| --- | --- | --- | --- | --- | --- | --- | --- | --- | --- |
|  | Case definition | Representativeness of cases | Selection of controls | Definition of controls | Study controls for age; gender/sex | Ascertainment of exposure | Same method of ascertainment for cases and controls | Non-response rate |  |
| Armon et al., 1991 ([7](#_ENREF_7)) | 1 | 0 | 0 | 1 | 2 | 0 | 1 | 0 | 5 |
| Cruz et al., 1999 ([8](#_ENREF_8)) | 1 | 1 | 1 | 1 | 2 | 0 | 1 | 0 | 7 |
| Muddasir Qureshi et al., 2006 ([9](#_ENREF_9)) | 1 | 0 | 1 | 1 | 2 | 0 | 1 | 0 | 6 |
| Furby et al., 2010 ([2](#_ENREF_2)) | 1 | 1 | 0 | 1 | 2 | 0 | 1 | 0 | 6 |
| Vinceti et al., 2010 ([35](#_ENREF_35)) | 1 | 1 | 1 | 0 | 2 | 1 | 1 | 0 | 7 |
| Das et al., 2012 ([10](#_ENREF_10)) | 1 | 0 | 1 | 1 | 2 | 0 | 1 | 0 | 6 |
| Pamphlett et Rikard-Bell, 2013 ([29](#_ENREF_29)) | 1 | 1 | 1 | 1 | 0 | 0 | 1 | 1 | 6 |
| Yu et al., 2014 ([11](#_ENREF_11)) | 1 | 0 | 1 | 1 | 2 | 0 | 1 | 0 | 6 |
| Malek et al., 2015 ([4](#_ENREF_4)) | 1 | 1 | 0 | 1 | 2 | 1 | 1 | 0 | 7 |
| Andrew et al., 2017 ([36](#_ENREF_36)) | 1 | 1 | 0 | 1 | 2 | 0 | 1 | 0 | 6 |
| Seelen et al., 2017 ([5](#_ENREF_5)) | 1 | 1 | 1 | 1 | 2 | 1 | 1 | 1 | 9 |
| Vinceti et al., 2017a ([12](#_ENREF_12)) | 1 | 1 | 1 | 0 | 2 | 1 | 1 | 1 | 8 |
| Vinceti et al., 2017b ([13](#_ENREF_13)) | 1 | 1 | 1 | 0 | 2 | 1 | 1 | 1 | 8 |
| Dickerson et al., 2018 ([30](#_ENREF_30)) | 0 | 1 | 1 | 0 | 2 | 1 | 1 | 1 | 7 |
| Povedano et al., 2018 ([14](#_ENREF_14)) | 1 | 1 | 0 | 1 | 2 | 1 | 1 | 1 | 8 |
| Peters et al., 2019 ([15](#_ENREF_15)) | 1 | 1 | 1 | 0 | 2 | 1 | 1 | 1 | 8 |
| Visser et al., 2019 ([31](#_ENREF_31)) | 1 | 1 | 1 | 1 | 2 | 1 | 1 | 0 | 8 |
| Filippini et al., 2020a ([16](#_ENREF_16)) | 1 | 1 | 1 | 0 | 2 | 0 | 1 | 0 | 6 |
| Filippini et al., 2020b ([37](#_ENREF_37)) | 1 | 1 | 1 | 0 | 2 | 0 | 1 | 0 | 6 |
| Fiore et al., 2020 ([38](#_ENREF_38)) | 1 | 1 | 1 | 1 | 2 | 1 | 1 | 1 | 9 |
| Bellavia et al., 2021 ([32](#_ENREF_32)) | 0 | 1 | 1 | 0 | 2 | 1 | 1 | 1 | 7 |
| Filippini et al., 2021 ([33](#_ENREF_33)) | 1 | 1 | 0 | 1 | 2 | 1 | 1 | 1 | 8 |
| Stipa et al., 2021 ([39](#_ENREF_39)) | 1 | 0 | 0 | 1 | 2 | 0 | 1 | 0 | 5 |
| Yu et al., 2021 ([34](#_ENREF_34)) | 1 | 1 | 1 | 0 | 2 | 1 | 1 | 1 | 8 |

See Supplementary Methods 2 for Newcastle-Ottawa quality assessment scale guide for case control studies.

**Table S6.** Newcastle-Ottawa risk quality assessment scores for the 4 cohort studies included in the conducted systematic reviews (n = 3)

| Reference | Selection | | | | Comparability | Outcome | | | Total |
| --- | --- | --- | --- | --- | --- | --- | --- | --- | --- |
|  | Representativeness of the exposed cohort | Selection of the non-exposed cohort | Ascertainment of exposure | Demonstration that outcome of interest was not present at start of study | Study controls for age; gender/sex | Assessment of outcome | Follow-up long enough for outcomes to occur | Adequacy of follow-up of cohorts |  |
| Vinceti et al., 1996 ([40](#_ENREF_40)) | 1 | 1 | 1 | 1 | 0 | 1 | 1 | 1 | 7 |
| Bove et al., 2014 ([41](#_ENREF_41)) | 0 | 0 | 1 | 0 | 1 | 1 | 1 | 1 | 5 |
| Vinceti et al., 2019 ([42](#_ENREF_42)) | 1 | 1 | 1 | 1 | 2 | 1 | 1 | 0 | 8 |
| Luna et al., 2019 ([17](#_ENREF_17)) | 1 | 1 | 1 | 1 | 2 | 1 | 1 | 0 | 8 |

See Supplementary Methods 3 for Newcastle-Ottawa quality assessment scale guide for cohort studies.

**Table S7.** Modified Newcastle-Ottawa risk of bias scores for the 2 cross-sectional studies included in the conducted systematic reviews (n = 3)

| Reference | Representativeness | Sample size | Non-respondents | Ascertainment | Descriptive statistics | Total |
| --- | --- | --- | --- | --- | --- | --- |
| Bettini et al., 2011 ([18](#_ENREF_18)) | 1 | 0 | 1 | 0 | 1 | 3 |
| Wei et al., 2015 ([19](#_ENREF_19)) | 1 | 1 | 1 | 0 | 1 | 4 |

See Supplementary Methods 4 for Modified Newcastle-Ottawa risk of bias scoring guide for cross-sectional studies.

**References**

1. Belter CW. Citation analysis as a literature search method for systematic reviews. Journal of the Association for Information Science and Technology. 2016;67(11):2766-77.

2. Furby A, Beauvais K, Kolev I, Rivain J-G, Sébille V. Rural environment and risk factors of amyotrophic lateral sclerosis: a case–control study. Journal of neurology. 2010;257(5):792-8.

3. Scott KM, Abhinav K, Wijesekera L, Ganesalingam J, Goldstein LH, Janssen A, et al. The association between ALS and population density: A population based study. Amyotroph Lateral Scler. 2010;11(5):435-8.

4. Malek AM, Barchowsky A, Bowser R, Heiman-Patterson T, Lacomis D, Rana S, et al. Exposure to hazardous air pollutants and the risk of amyotrophic lateral sclerosis. Environmental Pollution. 2015;197:181-6.

5. Seelen M, Toro Campos RA, Veldink JH, Visser AE, Hoek G, Brunekreef B, et al. Long-Term Air Pollution Exposure and Amyotrophic Lateral Sclerosis in Netherlands: A Population-based Case-control Study. Environ Health Perspect. 2017;125(9):097023.

6. Torbick N, Hession S, Stommel E, Caller T. Mapping amyotrophic lateral sclerosis lake risk factors across northern New England. International journal of health geographics. 2014;13(1):1.

7. Armon C, Kurland LT, Daube JR, O'Brien PC. Epidemiologic correlates of sporadic amyotrophic lateral sclerosis. Neurology. 1991;41(7):1077-.

8. Cruz DC, Nelson LM, McGuire V, Longstreth Jr W. Physical trauma and family history of neurodegenerative diseases in amyotrophic lateral sclerosis: a population-based case-control study. Neuroepidemiology. 1999;18(2):101-10.

9. Muddasir Qureshi M, Hayden D, Urbinelli L, Ferrante K, Newhall K, Myers D, et al. Analysis of factors that modify susceptibility and rate of progression in amyotrophic lateral sclerosis (ALS). Amyotrophic Lateral Sclerosis. 2006;7(3):173-82.

10. Das K, Nag C, Ghosh M. Familial, environmental, and occupational risk factors in development of amyotrophic lateral sclerosis. N Am J Med Sci. 2012;4(8):350-5.

11. Yu Y, Su F-C, Callaghan BC, Goutman SA, Batterman SA, Feldman EL. Environmental risk factors and amyotrophic lateral sclerosis (ALS): a case-control study of ALS in Michigan. PloS one. 2014;9(6):e101186.

12. Vinceti M, Malagoli C, Fabbi S, Kheifets L, Violi F, Poli M, et al. Magnetic fields exposure from high-voltage power lines and risk of amyotrophic lateral sclerosis in two Italian populations. Amyotrophic Lateral Sclerosis and Frontotemporal Degeneration. 2017;18(7-8):583-9.

13. Vinceti M, Filippini T, Violi F, Rothman KJ, Costanzini S, Malagoli C, et al. Pesticide exposure assessed through agricultural crop proximity and risk of amyotrophic lateral sclerosis. Environ Health. 2017;16(1):91.

14. Povedano M, Saez M, Martinez-Matos JA, Barcelo MA. Spatial Assessment of the Association between Long-Term Exposure to Environmental Factors and the Occurrence of Amyotrophic Lateral Sclerosis in Catalonia, Spain: A Population-Based Nested Case-Control Study. Neuroepidemiology. 2018;51(1-2):33-49.

15. Peters S, Visser AE, D’Ovidio F, Beghi E, Chiò A, Logroscino G, et al. Associations of electric shock and extremely low-frequency magnetic field exposure with the risk of amyotrophic lateral sclerosis: the Euro-MOTOR Project. American journal of epidemiology. 2019;188(4):796-805.

16. Filippini T, Tesauro M, Fiore M, Malagoli C, Consonni M, Violi F, et al. Environmental and occupational risk factors of amyotrophic lateral sclerosis: A population-based case-control study. International journal of environmental research and public health. 2020;17(8):2882.

17. Luna J, Leleu J-P, Preux P-M, Corcia P, Couratier P, Marin B, et al. Residential exposure to ultra high frequency electromagnetic fields emitted by Global System for Mobile (GSM) antennas and amyotrophic lateral sclerosis incidence: A geo-epidemiological population-based study. Environmental research. 2019;176:108525.

18. Bettini M, Gargiulo-Monachelli GM, Rodríguez G, Rey RC, Peralta LM, Sica REP. Epidemiology of amyotrophic lateral sclerosis patients in a centre in Buenos Aires. Arq Neuropsiquiatr. 2011;69(6):867-70.

19. Wei Q, Chen X, Zheng Z, Huang R, Guo X, Cao B, et al. Clinical features of amyotrophic lateral sclerosis in south-west China. Amyotrophic Lateral Sclerosis and Frontotemporal Degeneration. 2015;16(7-8):512-9.

20. Juvarra G, Bettoni L, Bortone E, Garavelli A, Montanari E, Rocca M. Amyotrophic lateral sclerosis in the province of Parma, Italy: a clinical and epidemiological study in the period 1960-1980. Ital J Neurol Sci. 1983;4:473-8.

21. Kalfakis N, Vassilopoulos D, Voumvourakis C, Ndjeveleka M, Papageorgiou C. Amyotrophic lateral sclerosis in Southern Greece: an epidemiological study. Neuroepidemiology. 1991;10:170-3.

22. Bettoni L, Bazzani M, Bortone E, Dascola I, Pisani E, Mancia D. Steadiness of amyotrophic lateral sclerosis in the province of Parma, Italy, 1960-1990. Acta Neurol Scand. 1994;90:276-80.

23. Mandrioli J, Fagliano P, Merelli E, Sola P. The epidemiology of ALS in Modena, Italy. Neurology. 2003;60:683-9.

24. Govoni V, Granieri E, Fallica E, Casetta I. Amyotrophic lateral sclerosis, rural environment and agricultural work in the Local Health District of Ferrara, Italy, in the years 1964-1998. J Neurol. 2005;252(11):1322-7.

25. Boumediene F, Druet-Cabanac M, Marin B, Preux PM, Allee P, Couratier P. Contribution of geolocalisation to neuroepidemiological studies: incidence of ALS and environmental factors in Limousin, France. J Neurol Sci. 2011;309(1-2):115-22.

26. Mandrioli J, Biguzzi S, Guidi C, Venturini E, Sette E, Terlizzi E, et al. Epidemiology of amyotrophic lateral sclerosis in Emilia Romagna Region (Italy): A population based study. Amyotroph Lateral Scler Frontotemporal Degener. 2014;15(3-4):262-8.

27. Rooney J, Heverin M, Vajda A, Crampsie A, Tobin K, Byrne S, et al. An exploratory spatial analysis of ALS incidence in Ireland over 17.5 years (1995-July 2013). PLoS One. 2014;9(5):e96556.

28. Rooney JP, Tobin K, Crampsie A, Vajda A, Heverin M, McLaughlin R, et al. Social deprivation and population density are not associated with small area risk of amyotrophic lateral sclerosis. Environ Res. 2015;142:141-7.

29. Pamphlett R, Rikard-Bell A. Different occupations associated with amyotrophic lateral sclerosis: is diesel exhaust the link? PLoS One. 2013;8(11):e80993.

30. Dickerson AS, Hansen J, Gredal O, Weisskopf MG. Amyotrophic Lateral Sclerosis and Exposure to Diesel Exhaust in a Danish Cohort. American Journal of Epidemiology. 2018;187(8):1613-22.

31. Visser AE, D'Ovidio F, Peters S, Vermeulen RC, Beghi E, Chiò A, et al. Multicentre, population-based, case–control study of particulates, combustion products and amyotrophic lateral sclerosis risk. Journal of Neurology, Neurosurgery & Psychiatry. 2019;90(8):854-60.

32. Bellavia A, Dickerson AS, Rotem RS, Hansen J, Gredal O, Weisskopf MG. Joint and interactive effects between health comorbidities and environmental exposures in predicting amyotrophic lateral sclerosis. International Journal of Hygiene and Environmental Health. 2021;231:113655.

33. Filippini T, Mandrioli J, Malagoli C, Costanzini S, Cherubini A, Maffeis G, et al. Risk of Amyotrophic Lateral Sclerosis and Exposure to Particulate Matter from Vehicular Traffic: A Case-Control Study. International Journal of Environmental Research and Public Health. 2021;18(3):973.

34. Yu Z, Peters S, van Boxmeer L, Downward GS, Hoek G, Kioumourtzoglou M-A, et al. Long-Term Exposure to Ultrafine Particles and Particulate Matter Constituents and the Risk of Amyotrophic Lateral Sclerosis. Environmental Health Perspectives. 2021;129(9):097702.

35. Vinceti M, Bonvicini F, Rothman KJ, Vescovi L, Wang F. The relation between amyotrophic lateral sclerosis and inorganic selenium in drinking water: a population-based case-control study. Environ Health. 2010;9:77.

36. Andrew AS, Caller TA, Tandan R, Duell EJ, Henegan PL, Field NC, et al. Environmental and occupational exposures and amyotrophic lateral sclerosis in New England. NeuroDegenerative Diseases. 2017;17(2-3):110-6.

37. Filippini T, Fiore M, Tesauro M, Malagoli C, Consonni M, Violi F, et al. Clinical and Lifestyle Factors and Risk of Amyotrophic Lateral Sclerosis: A Population-Based Case-Control Study. Int J Environ Res Public Health. 2020;17(3).

38. Fiore M, Parisio R, Filippini T, Mantione V, Platania A, Odone A, et al. Living near waterbodies as a proxy of cyanobacteria exposure and risk of amyotrophic lateral sclerosis: A population based case-control study. Environmental research. 2020;186:109530.

39. Stipa G, Ancidoni A, Mazzola M, Testai E, Funari E, Spera C, et al. Is Chronic Exposure to Raw Water a Possible Risk Factor for Amyotrophic Lateral Sclerosis? A Pilot Case-Control Study. Brain Sciences. 2021;11(2):193.

40. Vinceti M, Guidetti D, Pinotti M, Rovesti S, Merlin M, Vescovi L, et al. Amyotrophic Lateral Sclerosis after Long-Term Exposure to Drinking Water with High Selenium Content. Epidemiology. 1996;7(5):529-32.

41. Bove FJ, Ruckart PZ, Maslia M, Larson TC. Evaluation of mortality among marines and navy personnel exposed to contaminated drinking water at USMC base Camp Lejeune: a retrospective cohort study. Environmental Health. 2014;13(1):1-14.

42. Vinceti M, Filippini T, Malagoli C, Violi F, Mandrioli J, Consonni D, et al. Amyotrophic lateral sclerosis incidence following exposure to inorganic selenium in drinking water: A long-term follow-up. Environ Res. 2019;179(Pt A):108742.

43. Caller TA, Doolin JW, Haney JF, Murby AJ, West KG, Farrar HE, et al. A cluster of amyotrophic lateral sclerosis in New Hampshire: a possible role for toxic cyanobacteria blooms. Amyotroph Lateral Scler. 2009;10 Suppl 2:101-8.

44. Torbick N, Ziniti B, Stommel E, Linder E, Andrew A, Caller T, et al. Assessing Cyanobacterial Harmful Algal Blooms as Risk Factors for Amyotrophic Lateral Sclerosis. Neurotox Res. 2018;33(1):199-212.

45. Tesauro M, Bruschi M, Filippini T, D'Alfonso S, Mazzini L, Corrado L, et al. Metal (loid) s role in the pathogenesis of amyotrophic lateral sclerosis: Environmental, epidemiological, and genetic data. Environmental Research. 2021;192:110292.

46. Tesauro M, Consonni M, Filippini T, Mazzini L, Pisano F, Chiò A, et al. Incidence of amyotrophic lateral sclerosis in the province of Novara, Italy, and possible role of environmental pollution. Amyotrophic Lateral Sclerosis and Frontotemporal Degeneration. 2017;18(3-4):284-90.
